# The economic burden of asthma prior to death: a nationwide descriptive study

**DOI:** 10.3389/fpubh.2024.1191788

**Published:** 2024-02-19

**Authors:** Laurent Guilleminault, Michael Mounié, Agnès Sommet, Claire Camus, Alain Didier, Laurent Lionel Reber, Cécile Conte, Nadège Costa

**Affiliations:** ^1^Pôle des voies respiratoires, service de pneumo-allergologie, Centre Hospitalo-Universitaire de Toulouse, Toulouse, France; ^2^Toulouse Institute for Infectious and Inflammatory Diseases (Infinity), Inserm U1291, University of Toulouse, CNRS U5282, Toulouse, France; ^3^CRISALIS F-CRIN/INSERM, Toulouse, France; ^4^Unité d’Evaluation Médico-Economique, Centre Hospitalier Universitaire, Toulouse, France; ^5^INSERM-UMR 1295 - Center for Epidemiology and Research in POPulation health (CERPOP), Université de Toulouse, Inserm, UPS, Toulouse, France; ^6^Unité “Méthodologie, Data management, Analyses Statistiques”, Centre d’Investigation Clinique 1436, Service de pharmacologie médicale, Centre Hospitalo-Universitaire de Toulouse, Toulouse, France

**Keywords:** asthma, death, economic burden, SABA, costs, comorbidities

## Abstract

**Background:**

In addition to the clinical burden, asthma is responsible for a high economic burden. However, little is known about the economic burden of asthma prior to death.

**Objective:**

We performed an economic analysis to describe the costs during 12 and 24 months prior to asthma death between 2013 and 2017 in France.

**Methods:**

An observational cohort study was established using the French national health insurance database. Direct medical and non-medical costs, as well as costs related to absence from the workplace, were included in the analysis.

**Results:**

In total, 3,829 patients were included in the final analysis. Over 24 and 12 months prior to death, total medical costs per patient were €27,542 [26,545–28,641] and €16,815 [16,164–17,545], respectively. Total medical costs clearly increased over 24 months prior to death. Over 12 months prior to death, costs increased significantly according to age categories, with mean total costs of €8,592, €15,038, and €17,845, respectively, for the categories <18 years old, 18–75 years old, and 75+ years old (*p* < 0.0001). Over 12 months prior to death, costs were statistically higher in patients with a dispensation of six or more SABA canisters compared to those with a dispensation of five or less canisters (*p* < 0.0001). In multivariate analysis, comorbidities, hospital as location of death, and dispensation of 12 or more canisters of SABA per year are independent factors of the highest costs.

**Conclusion:**

To conclude, the economic burden of asthma death is high and increases with time, age, and SABA dispensation.

## Introduction

Asthma is a chronic respiratory disease that affects 340 million people worldwide (1). In addition to the clinical burden, asthma is responsible for a high economic burden. Over the past 10 years, this economic burden has been widely studied in different countries.

In a German study, the total annual costs per asthma patient were €2,168, with a €753 higher cost compared with a control group (2). In a Canadian study, the asthma cohort accrued, on average, an annual amount of €1,725 per patient in all-cause medical costs (3). The main cost component was medications, which accounted for 46.0% of additional costs compared to a sample of the general population. Outpatient visits and inpatient stays were responsible for 28.0 and 26.0% of additional costs compared to a sample of the general population, respectively. In France, asthma costs ranged from €393 for mild asthma to €3,187 for severe asthma (4). In the USA, during the 2008–2013 period, asthma was responsible for $50.3 billion in medical costs and $29 billion due to asthma-related mortality (5). The annual per-person incremental medical costs of asthma were $3,266 (in 2015 US dollars), mainly of which were attributable to medication ($1,830), medical visits ($640), inpatient stays ($529), and hospital-based outpatient visits ($176).

Patients with uncontrolled asthma had higher medical costs and decreased productivity (6–8). In patients with uncontrolled severe asthma, the medical cost attributed to asthma is even three times greater than the cost for patients with severe, controlled disease (9). Productivity is also highly impacted by uncontrolled, severe asthma (10). Comorbidities and systemic steroid-related complications also play a crucial role in the additional direct and indirect medical costs related to asthma (11, 12).

Although asthma mortality declined sharply until the mid-2000s, it appears that the mortality rate has been stagnating for several years (13–16). Recently, we showed that half the patients who died from asthma received inadequate ICS doses, and only a small proportion had access to biological therapies (17). Less than 15% were referred to a specialist, and lung function tests were performed in less than 30% of patients. To the best of our knowledge, no data have been published on the economic burden before asthma death. An economic analysis of healthcare consumption prior to asthma death would permit better consideration of the healthcare needed for asthma management. Moreover, mortality reduction with better asthma management could be associated with reduced or avoided cost components.

Our study aims to describe the economic burden during the 2-year period prior to asthma death between 2013 and 2017 in France.

## Population and methods

### Data sources

A retrospective observational cohort study was established using the French National Health Insurance Database (SNDS), which integrates the main French health databases (French health insurance inter-scheme information system, hospital data, and death registry). The cohort characteristics have been described previously (17). Briefly, in France, healthcare consumption data in the SNDS are collected from patients enrolled in mandatory health insurance (about 99% of the French population). The SNDS database provides a global overview of patients’ care pathways without any loss of follow-up in France.

Patients who died from asthma between 2013 and 2017 were identified according to the ICD-10 (International Classification of Diseases, 10th Revision) codes, J45 and J46. Asthma was the main cause of death on the death certificate. Duplicate patients and patients with no health consumption 2 years prior to death (*n* = 34) were excluded.

The study protocol was approved by the French National Ethics Committee (registration number TPS 578472). The protocol was also approved by the French Data Protection Authority (registration number 919303v1).

### Study measurements

#### Demographic and clinical data

In order to take into account the specificity of age categories, patients were divided into three categories according to age: 0–18, 18–75, and ≥ 75. We derived baseline comorbidities and Charlson Comorbidity Index (CCI) scores from an algorithm developed specifically for this claims database (18). It uses medical procedures, drugs, and hospital discharge diagnosis in addition to the long-term illness (LTI) scheme to identify comorbidities and the CCI score. Updated weights are applied for a better assessment of the CCI.

#### Healthcare resource consumption

Short-acting beta agonist (SABA) use was quantified as the number of canisters recorded within the past 12 months before death. To enable a comparison of different types and numbers of doses in the SABA canisters, a standardized SABA canister unit was defined as 150 doses. Patients were grouped by the number of SABA canisters recorded: no canisters [1; 2] and considered appropriate use [3–5], [6; 10], and ≥11 (19).

Inhaled corticosteroid (ICS) use included both monotherapy and fixed combinations within the last 12 months prior to death. The mean daily ICS dose (in budesonide equivalents) was defined according to GINA guidelines (20). In children aged 0–11 years, the daily ICS dose was divided into no or negligible dose (<100 μg), low dose (100–200 μg), medium dose (201–400 μg), and high dose (>400 μg). In teenagers (12–17 years old) and adults (≥18 years old), the daily ICS dose was divided into no or negligible dose (<200 μg), low dose (200–400 μg), medium dose (401–800 μg) and high dose (>800 μg). An inadequate daily dose was defined as a dose inferior to the lower limit of a low daily dose that cannot be considered as maintenance therapy.

#### Cost components

Costs were recorded and analyzed from the perspective of French health insurance. Direct medical (i.e., hospitalizations, medical and paramedical acts, medications, consultations, medical devices), and non-medical (i.e., transportation) costs as well as costs related to absence from the workplace (i.e., daily allowance) were included in the analysis. Unit prices correspond to the tariffs used by French healthcare insurance multiplied by the corresponding reimbursement rate.

### Statistical analysis

A descriptive analysis of the patients’ characteristics was performed. Qualitative variables were expressed as numbers and percentages. Quantitative variables were expressed as mean and standard deviations, or median and interquartile range (IQR) when relevant. The global costs of patients who died from asthma were described over 24 months prior to death using mean and bias-corrected and accelerated 95% bootstrapped confidence intervals. In addition, histograms summarizing cost evolution over 6-month periods, 24 months prior to death, were drawn, grouped or not by age categories. In order to focus on the period close to death, the rest of the analysis was performed over a 12-month period prior to death. The non-parametric Kruskal–Wallis test was used to assess cost differences between groups when needed. Generalized linear model regressions with gamma distribution and log link were implemented to assess adjusted cost differences between groups of SABA or ICS dispensation. Adjustment variables were age, gender, CCI, and place of death.

Statistical analyses were performed using R, version 4.2.1.

## Results

### Patients’ characteristics

From 1 January 2013 to 31 December 2017, 3,863 subjects were recorded with a leading cause of death related to asthma. After the exclusion of duplicate patients and patients with no health consumption 2 years prior to death, 3,829 patients were included in the final analysis. The patients’ characteristics were described previously. The main characteristics are indicated in Table 1.

**Table 1:** Characteristics of the study population for the entire cohort and by age group (*N* = 3,829).

Characteristics	Age ∈ (75; +) (*n* = 2,571)	Age ∈ (18–75) (*n* = 1,194)	Age ∈ (12–18) (*n* = 27)	Age ∈ (0; 12) (*n* = 37)
*Gender, n (%)*				
Women	1,826 (74.9)	633 (53.0)	12 (44.4)	13 (35.1)
*Age, years*				
Median (IQR)	87 [83–92]	59 [49–67]	15 [13–16]	6 [3–9]
*Year of death, n (%)*				
2013	503 (19.6)	243 (20.3)	5	4
2014	505 (19.6)	229 (19.1)	3	11
2015	522 (20.3)	251 (21.0)	6	9
2016	505 (19.6)	246 (20.6)	8	7
2017	536 (20.8)	225 (18.8)	5	6
*Charlson comorbidity index, n (%)*				
CCI = 0	542 (21.1)	662 (55.4)	25 (92.6)	34 (91.9)
CCI = 1	800 (31.1)	332 (27.8)	2 (7.4)	1 (2.7)
CCI = 2	760 (29.6)	132 (11.1)	0 (0)	1 (2.7)
CCI = 3	469 (18.2)	68 (5.7)	0 (0)	1 (2.7)
At least one ICS dispensation*, *n* (%)	1,692 (65.8)	744 (62.3)	22 (81.5)	27 (73.0)
daily ICS dose**, μg Median (IQR)	795.5 [394.2–7095.9]	699 [262.8–1353.5]	350.4 [168.6–591.3]	135.7 [65.7.5–2893.1]
ICS/LABA dispensation, *n* (%)	1,083 (42.1)	540 (45.2)	16 (59.2)	17 (45.9)
Dispensation of two or more SABA canisters, *n* (%)	1,420 (55.2)	814 (68.2)	25 (92.6)	30 (81.1)
Number of SABA canisters, median (IQR)	7 (3–14)	8 (3–8)	9.5 (5–12)	4 (2–9)
*Number of SABA canisters recorded, n (%)*				
0	937 (36.4)	277 (23.2)	1 (3.7)	3 (8.1)
≤2	366 (14.2)	178 (14.9)	3 (11.1)	12 (32.4)
3–6	310 (12.1)	154 (12.9)	4 (14.8)	11 (29.7)
6–12	415 (16.1)	213 (17.8)	9 (33.3)	6 (16.2)
>12	543 (21.1)	372 (31.2)	10 (37.0)	5 (13.5)

### Main costs over 24 and 12 months prior to death

**Figure 1:**
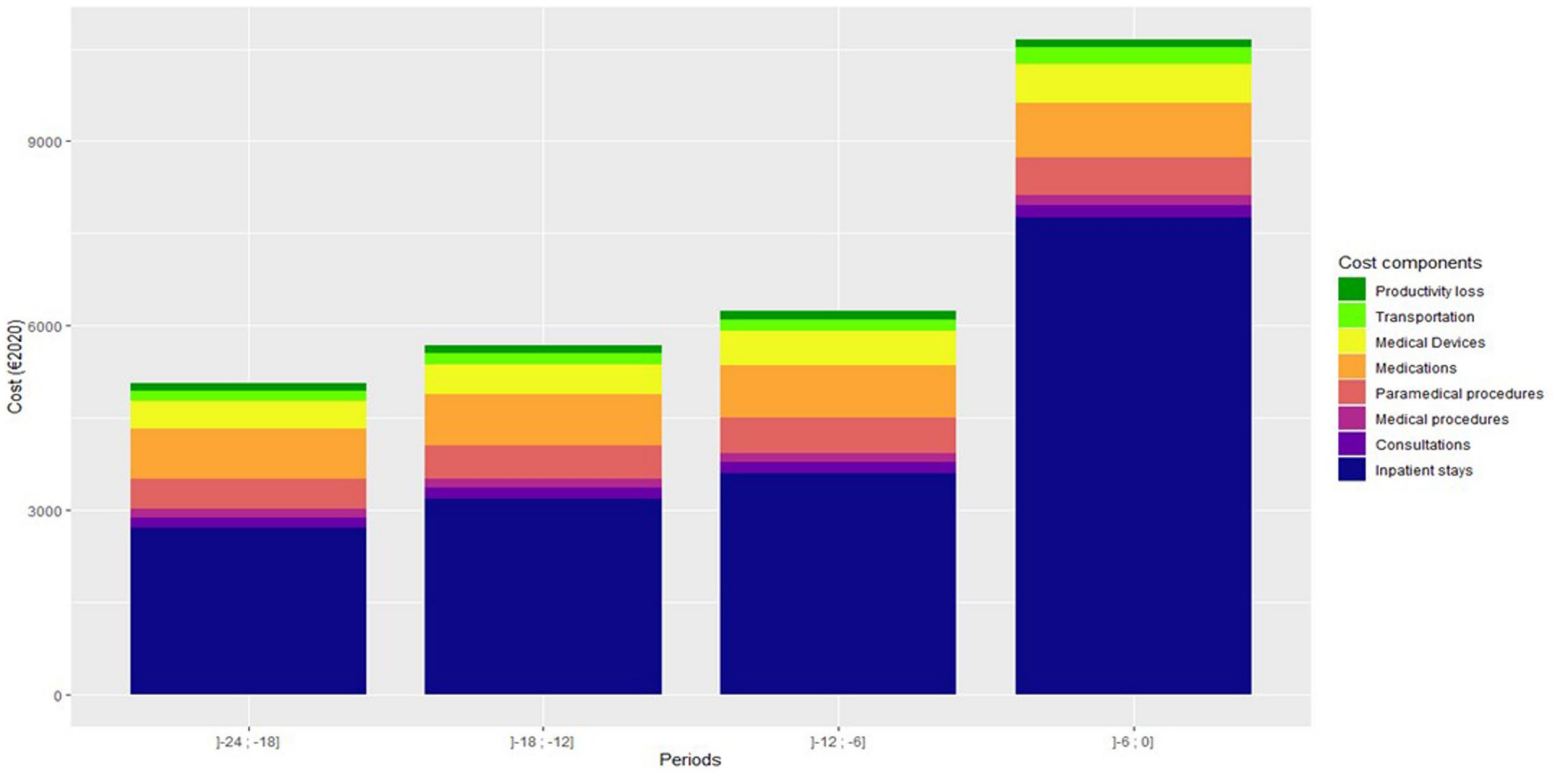
Direct and indirect costs of patients who died from asthma over 24 months prior to death.

Over 24 and 12 months prior to death, total medical costs per patient were €27,542 [26,545–28,641] and €16,815 [16,164–17,545], respectively (Table 2; Supplementary Table S1). The most important medical cost component was inpatient stays, regardless of the time prior to death (Figure 1; Table 2). Total medical costs clearly increased over 24 months prior to death and particularly during the last 6 months prior to death (Figure 1), notably due to death-related inpatient stays. Over 12 months prior to death, respiratory system medication accounted for 41% of the medication costs (Table 2).

**Table 2:** Main medical costs (€2020) of patients who died from asthma over 12 months prior to death.

	Period 1: −12; −6 months		Period 2: −6; 0 months		Period all: −12; −0 months	
	Frequency	Cost	Frequency	Cost	Frequency	Cost
Inpatient stays		3,561 [3,220; 4,037]		7,708 [7,348; 8,129]		11,270 [10,700; 11,898]
Medicine, surgery, obstetrics	0.7 [0.7; 0.9]	2,165 [2004; 2,358]	1.4 [1.3; 1.6]	6,121 [5,825; 6,478]	2.2 [2; 2.4]	8,286 [7,900; 8,715]
Respiratory system	0.2 [0.2; 0.2]	804 [709; 931]	0.6 [0.5; 0.6]	2,656 [2,481; 2,857]	0.8 [0.7; 0.8]	3,460 [3,218; 3,750]
Asthma-related	0.1 [0.1; 0.2]	466 [405; 566]	0.3 [0.3; 0.4]	1,311 [1,199; 1,440]	0.5 [0.4; 0.5]	1777 [1,632; 1951]
Rehabilitation	0.1 [0.1; 0.2]	1,069 [837; 1,490]	0.2 [0.2; 0.3]	1,243 [1,092; 1,423]	0.4 [0.3; 0.5]	2,311 [2023; 2,752]
Consultation	7.2 [6.8; 7.6]	181 [175; 188]	8.3 [7.9; 8.8]	212 [205; 218]	15.4 [14.7; 16.5]	392 [381; 405]
General practitioner	6.3 [6; 6.8]	133 [128; 137]	7.5 [7.1; 8]	156 [151; 161]	13.8 [13.1; 14.7]	288 [279; 298]
Pulmonologist	0.1 [0.1; 0.1]	2 [1; 2]	0.1 [0.1; 0.1]	2 [1; 2]		
Medical procedures	6.5 [6.3; 6.8]	154 [145; 165]	6.8 [6.6; 7.1]	158 [150; 166]	13.4 [12.9; 13.9]	312 [297; 328]
Paramedical procedures	37.2 [34.2; 41.6]	575 [536; 620]	37.7 [35; 41]	617 [573; 666]	74.9 [69.6; 82.5]	1,192 [1,116; 1,279]
Medications	83 [80.9; 85.2]	856 [813; 913]	87.1 [84.9; 89.3]	883 [841; 931]	170.1 [165.9; 174.2]	1739 [1,657; 1835]
Respiratory system	17 [16.3; 17.7]	358 [331; 393]	17.6 [16.9; 18.2]	359 [332; 393]	34.6 [33.3; 35.8]	717 [664; 780]
Medical device	5.5 [5.2; 5.7]	550 [516; 587]	6.6 [6.3; 6.9]	640 [606; 676]	12 [11.5; 12.6]	1,190 [1,125; 1,256]
Respiratory assistance devices, home oxygen therapy	1.2 [1.1; 1.3]	288 [263; 315]	1.4 [1.3; 1.5]	299 [275; 322]	2.6 [2.4; 2.8]	587 [542; 636]
Transportation	1.7 [1.6; 1.8]	195 [177; 221]	2.4 [2.2; 2.5]	263 [242; 290]	4 [3.8; 4.3]	458 [422; 505]
Productivity loss	1.1 [0.8; 1.6]	134 [109; 171]	1.1 [0.8; 1.5]	128 [104; 155]	2.2 [1.7; 3]	262 [214; 321]
Global	**143.1 [138.5; 148.7]**	**6,206 [5,835; 6,687]**	**151.7 [147.1; 156.5]**	**10,609 [10,189; 11,039]**	**294.8 [286; 304.9]**	**16,815 [16,164; 17,545]**

Data are expressed in mean [95% CI]. Global costs are indicated in bold.

### Costs according to age categories over 24 and 12 months prior to death

**Figure 2:**
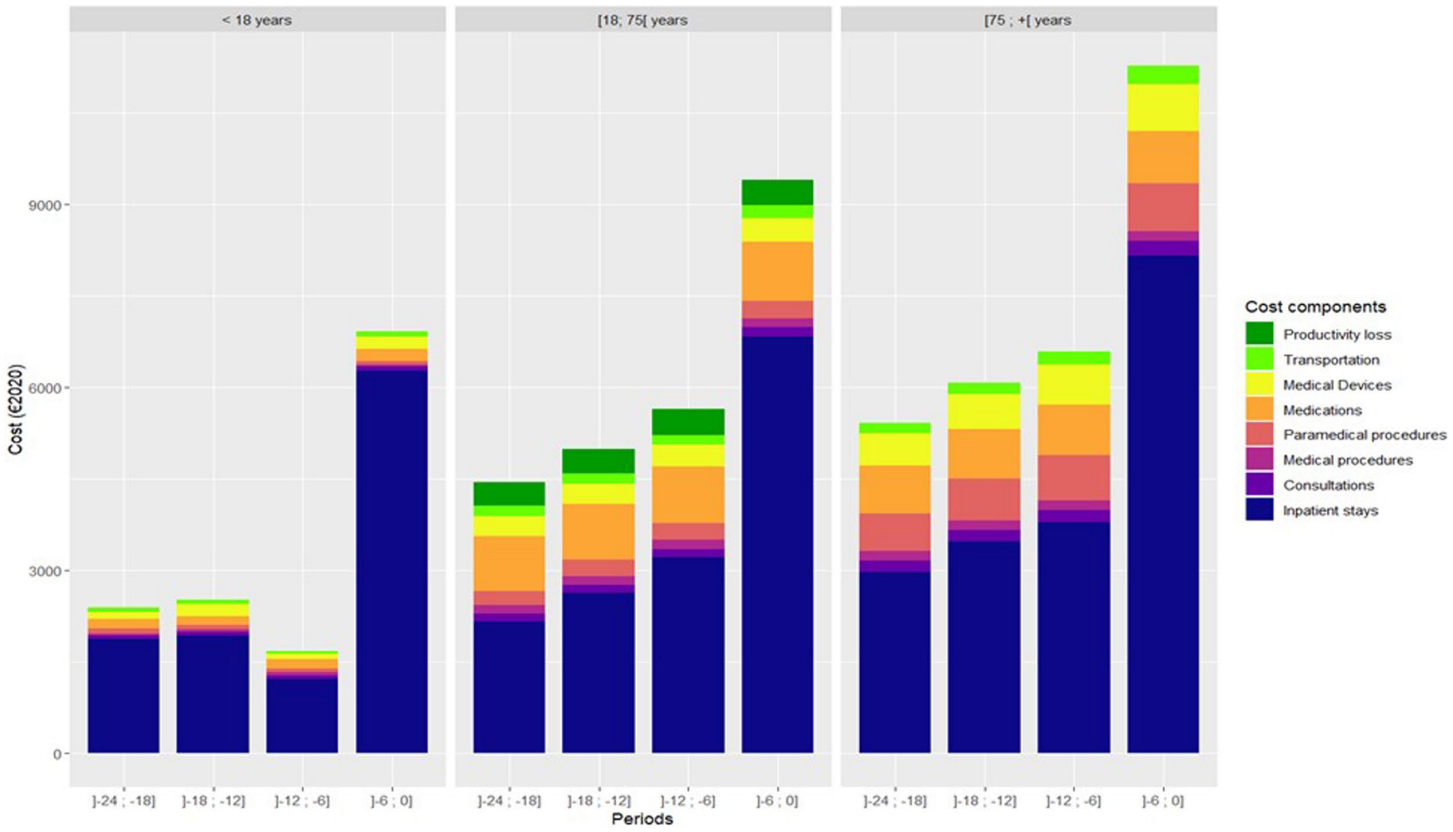
Direct and indirect costs of patients who died from asthma over 12 months prior to death according to age categories.

Over 24 and 12 months prior to death, it is noticeable that costs increased significantly according to age categories. The mean total costs were €8,592, €15,038, and €17,845 over 12 months prior to death, respectively, for the categories <18 years old, 18–75 years old, and 75+ years old (*p* < 0.0001) (Figure 2; Supplementary Table S2). The cost of rehabilitation care, paramedical procedures, medical devices, consultation, and transportation were significantly higher in patients aged 75+ years old compared to the others (Supplementary Table S2). However, the costs of respiratory system medications were significantly higher in patients aged 18–75 years old compared to the two other groups (*p* < 0.0001). In addition, 18- to 75-year-old patients are associated with productivity loss costs amounting to €840.

### Costs according to ICS dispensation over 12 months prior to death (overall population and age categories)

Over 12 months prior to death, total medical costs increased slightly with the daily dose of ICS in the whole population (Supplementary Table S3). Moreover, costs for consultations, medical and paramedical procedures, and medical devices were statistically higher in patients with medium or high daily doses of ICS compared to patients with no or low doses of ICS (*p* < 0.0001). In patients aged 18–75 years old, total medical costs were statistically lower in those with a dispensation of no or low daily dose of ICS compared to those with a medium and high daily dose of ICS (€13,244 and €13,533 vs. €18,704 and €16,856, respectively, *p* < 0.0001) (Table 3). A statistical difference was observed for all main cost components except for inpatient stays not related to the respiratory system between the categories of ICS daily dose.

**Table 3:** Main medical costs (€2020) according to age and ICS dispensation in patients who died from asthma over 12 months prior to death.

		0^a^ (*n* = 35)		Low^b^ (*n* = 6)		Moderate^c^ (*n* = 13)		High^d^ (*n* = 10)		
		Frequency	Cost	Frequency	Cost	Frequency	Cost	Frequency	Cost	*p*
<18	Inpatient stays	1.9 [1.2; 3.9]	6,556 [4,316; 9,663]	2.5 [1.2; 5]	16,136 [4,215; 43,116]	1.6 [1.1; 2.3]	5,601 [3,182; 8,584]	1.4 [0.8; 1.9]	7,962 [2,759; 17,632]	0.553
	Medicine, surgery, obstetrics	1.9 [1.1; 3.8]	6,325 [4,171; 9,341]	2.5 [1.2; 4.8]	16,045 [4,036; 39,079]	1.5 [0.9; 2.2]	5,352 [3,040; 8,636]	1.4 [0.8; 1.9]	7,851 [2,674; 16,965]	0.556
	Respiratory system	0.7 [0.4; 1.3]	3,567 [2001; 5,867]	1.2 [0.3; 2]	2,354 [675; 5,676]	1 [0.6; 1.8]	4,088 [1760; 7,667]	1 [0.6; 1.5]	4,209 [1,499; 13,474]	0.374
	Asthma-related	0.6 [0.3; 1.1]	2,373 [1,108; 4,649]	0.7 [0; 1.2]	1,657 [273; 3,896]	0.7 [0.3; 1]	1,655 [640; 3,271]	0.8 [0.3; 1.1]	3,864 [1,094; 12,528]	0.33
	Not related to the respiratory system	1.2 [0.5; 3.4]	2,758 [1,398; 5,267]	1.3 [0.5; 2.5]	13,692 [3,955; 36,892]	0.5 [0.2; 1.1]	1,264 [412; 3,849]	0.4 [0.1; 0.8]	3,642 [270; 14,758]	0.135
	Consultation	6 [4.3; 8.1]	126 [93; 169]	7.2 [3.8; 10.5]	158 [77; 218]	12.2 [7.7; 26.3]	184 [135; 279]	5.9 [3.3; 8.9]	121 [69; 193]	0.211
	General practitioner	1.1 [0.6; 1.9]	48 [22; 103]	2.2 [0.8; 3.7]	128 [47; 340]	3.1 [1.5; 5.5]	155 [83; 270]	0.3 [0; 0.5]	29 [7; 67]	0.014
	Medical procedures	5.8 [2.5; 12]	110 [50; 248]	2.8 [0.2; 7.3]	38 [0; 98]	6.3 [2; 13.4]	130 [33; 306]	15.7 [0; 62.4]	221 [0; 883]	0.671
	Paramedical procedures	39.9 [29; 54.6]	295 [194; 486]	28 [20.7; 35.2]	148 [103; 190]	50.5 [36.3; 65.3]	438 [285; 827]	58.3 [33.7; 93.3]	604 [316; 1,101]	0.088
	Medications	14.8 [9.9; 21.9]	126 [76; 196]	17.2 [14.1; 19.8]	119 [78; 176]	25.1 [17.3; 34.2]	234 [156; 315]	36.5 [18.8; 74.6]	543 [258; 1,032]	0.011
	Respiratory system	2.9 [1.1; 11.8]	445 [139; 1,529]	0.8 [0; 1.5]	10 [2; 29]	3.4 [1.8; 7]	82 [32; 209]	3.3 [0.6; 7.7]	62 [6; 175]	0.358
	Medical devices	0.4 [0.2; 0.9]	36 [3; 139]	0.7 [0; 1.2]	5 [1; 8]	0.6 [0.2; 1.2]	8 [2; 19]	0.1 [0; 0.3]	1 [0; 3]	0.31
	Respiratory assistance devices, home oxygen therapy	0.1 [0; 0.2]	0 [0; 1]	0.2 [0; 0.3]	5 [0; 10]	1.5 [0.2; 4]	50 [1; 155]	2.4 [0.2; 5.7]	45 [3; 136]	0.06
	Transportation	2.4 [0.5; 9.9]	230 [79; 561]	0.3 [0; 0.7]	11 [0; 22]	0.2 [0; 0.6]	76 [0; 198]	0.2 [0; 0.4]	14 [0; 35]	0.997
	Productivity loss	0 [0; 0]	0 [0; 0]	0 [0; 0]	0 [0; 0]	0 [0; 0]	0 [0; 0]	0 [0; 0]	0 [0; 0]	—
	Global		**7,810 [5,319; 11,834]**		**16,629 [5,110; 43,510]**		**6,666 [4,329; 9,735]**		**9,012 [3,292; 19,351]**	**0.733**

		0^a^ (*n* = 587)		Low^b^ (*n* = 108)		Moderate^c^ (*n* = 167)		High^d^ (*n* = 332)		
18–75	Inpatient stays		8,999 [7,406; 11,496]		9,194 [6,530; 13,797]		13,468 [10,555; 17,892]		10,411 [8,764; 12,472]	0.001
	Medicine, surgery, obstetrics	2.1 [1.5; 3.5]	7,059 [5,751; 8,715]	1.3 [0.9; 1.8]	7,265 [5,294; 11,091]	1.9 [1.5; 2.5]	10,154 [8,015; 13,227]	2.8 [2; 4.5]	9,001 [7,605; 10,782]	<0.001
	Respiratory system	0.4 [0.4; 0.5]	2,300 [1788; 3,596]	0.5 [0.3; 0.8]	3,678 [2,115; 7,619]	0.8 [0.6; 1.1]	4,912 [3,596; 6,865]	0.8 [0.6; 1]	4,156 [3,158; 5,513]	<0.001
	Asthma-related	0.3 [0.2; 0.4]	1,371 [968; 2,239]	0.4 [0.2; 0.6]	1,673 [1,008; 2,647]	0.5 [0.4; 0.7]	2,238 [1,562; 3,251]	0.6 [0.5; 0.8]	2,436 [1803; 3,341]	<0.001
	Not related to the respiratory system	1.7 [1; 3]	4,760 [3,757; 6,157]	0.7 [0.5; 1.3]	3,587 [2,463; 5,013]	1.1 [0.8; 1.7]	5,242 [3,714; 7,403]	2 [1.3; 3.8]	4,845 [3,889; 6,154]	0.124
	Consultation	11.1 [10; 13.2]	251 [229; 277]	10.1 [8.2; 13.6]	228 [195; 274]	12.2 [10.6; 14.2]	322 [279; 373]	15 [13.1; 17.4]	371 [334; 430]	<0.001
	General practitioner	8.4 [7.4; 10]	249 [216; 306]	7.2 [5.6; 9.9]	256 [183; 425]	11.9 [9.8; 14.6]	399 [317; 559]	10.8 [9.3; 12.7]	364 [307; 447]	0.001
	Medical procedures	32.9 [25.2; 42.5]	486 [356; 827]	45.8 [13.9; 161.1]	511 [230; 1,066]	39.5 [27.8; 57.4]	777 [491; 1,257]	60.3 [41.2; 94]	552 [424; 725]	<0.001
	Paramedical procedures	127 [115.8; 140.1]	1,435 [1,240; 1718]	122.5 [104.7; 146.6]	1895 [1,125; 3,906]	156.3 [138.2; 178.3]	1796 [1,478; 2,364]	200.1 [182.5; 219.9]	2,799 [2,385; 3,374]	<0.001
	Medications	27.1 [24.4; 30.4]	420 [365; 505]	31.8 [26.2; 39.6]	767 [405; 2,115]	38.7 [33.8; 45.5]	659 [548; 888]	56.8 [51.7; 64.2]	1,686 [1,355; 2,220]	<0.001
	Respiratory system	6.3 [5.4; 7.7]	589 [469; 729]	5.5 [3.9; 8.9]	436 [222; 1,161]	9.4 [7.4; 11.6]	935 [682; 1,287]	10.8 [9.3; 12.6]	1,010 [782; 1,243]	<0.001
	Medical devices	1.5 [1.1; 2.1]	290 [217; 388]	0.6 [0.2; 1.5]	161 [44; 544]	2.1 [1.3; 3.2]	503 [305; 763]	2.2 [1.7; 2.8]	529 [385; 705]	<0.001
	Respiratory assistance devices, home oxygen therapy	1.4 [1; 1.9]	36 [27; 51]	1.8 [1.2; 2.7]	48 [30; 72]	2.4 [1.7; 3.3]	66 [46; 102]	3.4 [2.8; 4.1]	72 [59; 90]	<0.001
	Transportation	2.5 [2; 3.2]	366 [262; 569]	1.5 [0.9; 2.4]	143 [79; 260]	3.5 [2.6; 4.7]	375 [261; 565]	3.1 [2.5; 3.9]	462 [311; 779]	<0.001
	Productivity loss	7.7 [5; 11.8]	868 [656; 1,179]	3.1 [0.9; 10.2]	870 [443; 1,651]	6.1 [2.9; 13.1]	632 [376; 1,052]	8 [4.9; 13.4]	886 [624; 1,244]	0.958
	Global		**13,244 [11,325; 16,342]**		**13,533 [10,047; 20,428]**		**18,704 [15,435; 23,304]**		**16,856 [14,728; 19,011]**	**<0.001**

		0^a^ (*n* = 1,141)		Low^b^ (*n* = 215)		Moderate^c^ (*n* = 375)		High^d^ (*n* = 840)		
>75	Inpatient stays		11,229 [10,366; 12,294]		13,978 [11,518; 17,511]		12,398 [10,961; 14,164]		12,173 [11,145; 13,446]	0.102
	Medicine, surgery, obstetrics	2 [1.8; 2.5]	7,738 [7,226; 8,319]	2.1 [1.8; 2.4]	8,671 [7,345; 10,292]	2.5 [2.1; 3.7]	9,064 [8,051; 10,340]	2.3 [2; 3.2]	8,996 [8,300; 9,805]	0.034
	Respiratory system	0.7 [0.7; 0.8]	2,946 [2,630; 3,317]	0.9 [0.8; 1.1]	3,934 [3,130; 5,213]	1 [0.8; 1.2]	3,950 [3,320; 4,846]	1 [0.9; 1]	4,021 [3,621; 4,566]	<0.001
	Asthma-related	0.4 [0.3; 0.4]	1,438 [1,241; 1709]	0.5 [0.4; 0.7]	1932 [1,456; 2,595]	0.7 [0.5; 0.8]	2,147 [1713; 2,659]	0.5 [0.5; 0.6]	1929 [1,680; 2,247]	<0.001
	Not related to the respiratory system	1.3 [1.1; 1.8]	4,792 [4,360; 5,292]	1.1 [0.9; 1.4]	4,736 [3,867; 5,815]	1.5 [1.1; 2.8]	5,114 [4,381; 6,148]	1.3 [1.1; 2.3]	4,976 [4,450; 5,546]	0.964
	Consultation	16 [14.7; 18.5]	411 [390; 434]	17.3 [14.5; 24.6]	442 [398; 501]	21 [18.3; 26.4]	495 [454; 540]	16.9 [15.7; 18.3]	471 [447; 494]	<0.001
	General practitioner	14.7 [13.7; 15.5]	298 [275; 324]	14.6 [12.7; 16.6]	298 [256; 368]	16.5 [15; 17.9]	328 [297; 363]	16.4 [15.4; 17.5]	359 [332; 396]	<0.001
	Medical procedures	79.6 [71.7; 89]	1,410 [1,265; 1,560]	101.8 [68.1; 251.6]	1,375 [1,093; 1789]	111 [88.9; 137.7]	1,638 [1,340; 1975]	96.7 [85.4; 111.3]	1,650 [1,468; 1883]	0.002
	Paramedical procedures	160.7 [153.9; 167.5]	1,406 [1,300; 1,562]	177.7 [164.3; 193.1]	1,392 [1,257; 1,569]	194.4 [183.1; 206.8]	1936 [1,688; 2,375]	206.8 [199; 215.7]	2057 [1937; 2,237]	<0.001
	Medications	24.4 [22.5; 26.3]	515 [444; 657]	29.5 [26.2; 33.7]	485 [423; 587]	36.2 [32.9; 40.3]	695 [572; 986]	46.1 [43.4; 49.1]	927 [865; 1,004]	<0.001
	Respiratory system	12.7 [11.7; 13.6]	1,350 [1,236; 1,484]	13.5 [11.5; 16.2]	1,285 [1,045; 1,600]	14 [12.6; 15.6]	1,355 [1,173; 1,598]	16.4 [15.1; 17.8]	1,584 [1,436; 1758]	0.002
	Medical devices	3.1 [2.7; 3.5]	684 [597; 780]	2.5 [1.9; 3.5]	561 [401; 780]	2.8 [2.3; 3.4]	687 [540; 867]	3.3 [2.9; 3.8]	761 [657; 873]	0.104
	Respiratory assistance devices, home oxygen therapy	1.8 [1.5; 2.1]	46 [38; 56]	2.4 [1.8; 3.2]	62 [45; 86]	3.1 [2.5; 3.7]	71 [56; 91]	3.6 [3.2; 4.2]	81 [70; 97]	<0.001
	Transportation	4.7 [4.3; 5.1]	490 [436; 585]	4.8 [4.1; 5.5]	482 [393; 589]	4.6 [4.1; 5.2]	506 [417; 825]	4.9 [4.5; 5.3]	531 [476; 620]	0.192
	Productivity loss	0 [0; 0]	0 [0; 0]	0 [0; 0]	0 [0; 0]	0 [0; 0]	0 [0; 0]	0 [0; 0]	0 [0; 1]	0.56
	Global		**16,593 [15,550; 17,756]**		**19,252 [16,448; 23,020]**		**18,656 [16,899; 20,641]**		**18,824 [17,543; 20,198]**	**<0.001**

Global costs are indicated in bold.

### Costs according to SABA dispensation (overall population and age categories)

Over 12 months prior to death, medical costs tend to increase with the number of SABA dispensation in the whole population (Table 4). Costs were statistically higher in patients with a dispensation of six or more canisters compared to those with a dispensation of five or less canisters (*p* < 0.0001). The cost difference was not restricted to medication and was notably led by inpatient stay costs. In addition, significant cost increases were found for patients with more than 12 dispensations of canisters for medical and paramedical procedures, consultations, medical devices, and productivity loss.

**Table 4:** Main medical costs (€2020) according to age and SABA dispensation in patients who died from asthma.

		0 Canister (*n* = 12)		[1; 2] Canisters (*n* = 14)		[3; 5] Canisters (*n* = 8)		[6; 12] Canisters (*n* = 16)		>12 Canisters (*n* = 14)		
		Frequency	Cost	Frequency	Cost	Frequency	Cost	Frequency	Cost	Frequency	Cost	*p*
<18	Inpatient stays	2.9 [1.2; 8.9]	9,387 [5,288; 15,487]	1.1 [0.6; 1.8]	3,557 [1,656; 6,661]	2.5 [1.2; 4.2]	11,177 [5,611; 19,971]	1.8 [1; 3.3]	10,007 [4,904; 22,744]	1.2 [0.7; 1.9]	4,767 [2,331; 11,050]	0.274
	Medicine, surgery, obstetrics	2.9 [1.2; 8.6]	9,150 [4,857; 14,990]	1.1 [0.6; 1.8]	3,339 [1,455; 6,201]	2.5 [1.2; 4.1]	11,017 [5,621; 20,487]	1.8 [1; 3.3]	9,872 [4,834; 21,121]	1.1 [0.6; 1.8]	4,507 [1954; 10,308]	0.178
	Respiratory system	0.9 [0.4; 1.5]	5,758 [2,832; 9,364]	0.4 [0.1; 0.8]	1823 [267; 5,238]	0.6 [0.1; 0.9]	3,152 [781; 7,522]	1.1 [0.5; 2.1]	3,464 [1,430; 7,734]	1.1 [0.6; 1.8]	4,211 [1830; 10,210]	0.389
	Asthma-related	0.8 [0.2; 1.3]	3,270 [1,326; 6,734]	0.4 [0.1; 0.6]	1,204 [215; 4,901]	0.4 [0; 0.6]	1884 [139; 7,074]	0.9 [0.4; 1.7]	2,508 [1,024; 9,203]	0.7 [0.4; 1.1]	2,989 [947; 10,378]	0.668
	Not related to the respiratory system	2 [0.2; 7.4]	3,392 [358; 9,793]	0.7 [0.4; 1]	1,516 [755; 3,759]	1.9 [0.9; 3.6]	7,864 [3,464; 18,380]	0.7 [0.3; 1.4]	6,409 [2,205; 16,841]	0.1 [0; 0.2]	296 [3; 1,340]	0.020
	Consultation	6.1 [3.8; 9.2]	137 [90; 224]	3.5 [1.8; 6.4]	84 [42; 152]	17.6 [10.5; 34.3]	218 [159; 313]	6.7 [4.8; 10.1]	149 [101; 224]	7.1 [5; 9.8]	144 [101; 200]	0.040
	General practitioner	2 [0.8; 3.9]	79 [32; 155]	0.5 [0.1; 1.4]	18 [4; 49]	0.6 [0.1; 1]	30 [11; 49]	2.1 [1; 4.3]	134 [59; 243]	1.9 [0.7; 3.5]	84 [37; 208]	0.169
	Medical procedures	7.6 [1.9; 21]	162 [35; 550]	4 [0.4; 11.2]	81 [8; 233]	23.8 [2.5; 81]	402 [54; 1,229]	7.5 [2.9; 17.9]	105 [40; 232]	0.1 [0; 0.3]	1 [0; 2]	0.273
	Paramedical procedures	55.7 [33.8; 88.3]	440 [225; 787]	17.5 [11.7; 25.7]	95 [57; 140]	43.9 [28.4; 68.3]	268 [140; 452]	40.4 [29.9; 58.7]	452 [274; 807]	64 [43; 86.1]	496 [288; 882]	0.023
	Medications	14.7 [8.1; 21.9]	126 [65; 195]	7.9 [4.9; 13]	59 [32; 104]	12.4 [9; 18.3]	98 [39; 288]	22.1 [16.1; 29.4]	278 [184; 405]	41 [26.1; 64.9]	430 [221; 857]	0.005
	Respiratory system	6.5 [1.4; 25.6]	869 [24; 3,935]	0.7 [0.3; 1.2]	132 [5; 725]	2.8 [1.5; 4.1]	105 [27; 390]	1.9 [0.7; 5.2]	93 [13; 260]	3.1 [1; 6.4]	195 [44; 759]	0.265
	Medical devices	0.2 [0; 0.3]	2 [0; 6]	0.2 [0; 0.4]	2 [0; 5]	1.2 [0.5; 2.2]	58 [6; 256]	0.7 [0.2; 1.4]	55 [3; 254]	0.1 [0; 0.4]	2 [0; 6]	0.040
	Respiratory assistance devices, home oxygen therapy	0.3 [0; 0.7]	3 [0; 6]	0 [0; 0]	0 [0; 0]	0.2 [0; 0.8]	4 [0; 12]	0.7 [0; 2.6]	30 [0; 117]	2.1 [0.6; 4.9]	43 [10; 105]	0.513
	Transportation	1.8 [0.1; 4.6]	338 [30; 1,020]	4.4 [0; 17.3]	193 [0; 759]	0.9 [0.2; 1.8]	243 [17; 652]	0 [0; 0]	0 [0; 0]	0.2 [0; 0.4]	39 [4; 152]	0.175
	Productivity loss	0 [0; 0]	0 [0; 0]	0 [0; 0]	0 [0; 0]	0 [0; 0]	0 [0; 0]	0 [0; 0]	0 [0; 0]	0 [0; 0]	0 [0; 0]	—
	Global		**11,410 [6,448; 21,693]**		**4,159 [2,201; 7,193]**		**12,443 [6,300; 21,863]**		**10,940 [5,643; 20,359]**		**5,726 [3,217; 11,350]**	0.185

		0 Canister (*n* = 316)		[1; 2] Canisters (*n* = 191)		[3; 5] Canisters (*n* = 140)		[6; 12] Canisters (*n* = 214)		>12 Canisters (*n* = 333)		
18–75	Inpatient stays	2 [1.4; 3.9]	8,193 [6,370; 12,377]	2.4 [1.6; 5.7]	9,233 [6,451; 14,576]	1.6 [1.1; 2.7]	7,925 [5,676; 12,352]	2.2 [1.7; 2.9]	10,576 [8,424; 13,479]	3.9 [2.6; 6.4]	12,781 [10,770; 15,043]	<0.001
	Medicine, surgery, obstetrics	1.5 [1.1; 2.4]	5,925 [4,764; 7,477]	2.1 [1.2; 5.1]	8,013 [5,473; 12,890]	1.4 [1; 2.2]	5,658 [4,052; 8,150]	1.7 [1.4; 2.2]	8,021 [6,463; 9,975]	3.5 [2.4; 5.8]	11,114 [9,357; 13,222]	<0.001
	Respiratory system	0.4 [0.3; 0.5]	2,432 [1776; 3,913]	0.4 [0.3; 0.5]	2,714 [1,475; 6,986]	0.6 [0.4; 0.9]	2,251 [1,480; 3,633]	0.6 [0.5; 0.8]	3,151 [2,250; 4,589]	0.9 [0.7; 1.1]	5,018 [4,004; 6,485]	<0.001
	Asthma-related	0.2 [0.2; 0.3]	961 [656; 1,492]	0.3 [0.2; 0.4]	1,678 [796; 4,576]	0.4 [0.3; 0.6]	1,189 [761; 1988]	0.4 [0.3; 0.6]	1797 [1,222; 2,592]	0.7 [0.5; 0.9]	2,981 [2,320; 3,892]	<0.001
	Not related to the respiratory system	1.1 [0.8; 2]	3,493 [2,638; 4,567]	1.7 [0.8; 4.4]	5,299 [3,405; 10,327]	0.9 [0.5; 1.6]	3,407 [2,269; 5,130]	1.2 [0.9; 1.7]	4,870 [3,664; 6,488]	2.6 [1.4; 5]	6,096 [4,873; 7,650]	<0.001
	Consultation	11.7 [10; 15.7]	246 [221; 277]	10.2 [8.9; 11.9]	260 [226; 314]	11 [9.1; 13.4]	281 [234; 365]	11.3 [9.7; 13.5]	253 [221; 296]	15 [13.3; 17.2]	384 [346; 443]	<0.001
	General practitioner	8.7 [7.5; 10.4]	256 [211; 325]	9.5 [7.7; 14.4]	289 [232; 373]	8.3 [6.7; 10.6]	321 [233; 539]	7.8 [6.6; 9.4]	260 [211; 339]	11.5 [10; 13.9]	375 [317; 479]	0.003
	Medical procedures	49.3 [31; 89.1]	590 [400; 866]	35.6 [23.4; 55]	455 [309; 760]	21.9 [12.8; 36.8]	372 [212; 678]	31 [20; 50.2]	350 [234; 547]	56.5 [41.2; 83.5]	761 [535; 1,272]	<0.001
	Paramedical procedures	124.3 [110.2; 140.2]	1,579 [1,280; 2,258]	131.4 [114; 150.2]	1,400 [1,152; 1787]	127.8 [107.5; 155.7]	1763 [1,307; 2,772]	131.3 [115.6; 149.9]	1,620 [1,263; 2,332]	210 [191.6; 233.7]	2,752 [2,348; 3,280]	<0.001
	Medications	21 [18; 24.2]	419 [324; 689]	25.4 [20.7; 31.8]	497 [370; 794]	29.2 [25.3; 34.6]	572 [453; 887]	34.8 [30.7; 40]	898 [590; 1,630]	64.9 [59.6; 72.3]	1,500 [1,204; 1952]	<0.001
	Respiratory system	6.2 [5; 7.7]	613 [458; 854]	7 [5.6; 9]	604 [420; 877]	6.3 [4.6; 8.7]	691 [444; 1,127]	6.8 [5.5; 8.5]	581 [430; 811]	11.5 [9.8; 13.7]	1,063 [855; 1,311]	<0.001
	Medical devices	1.5 [1; 2.2]	329 [219; 488]	0.9 [0.5; 1.5]	157 [82; 316]	1.3 [0.8; 2.2]	356 [188; 613]	1.6 [1.1; 2.5]	307 [194; 492]	2.6 [2; 3.4]	595 [447; 781]	<0.001
	Respiratory assistance devices, home oxygen therapy	1 [0.7; 1.6]	19 [13; 29]	1.5 [1; 2.4]	32 [21; 55]	1.3 [0.8; 2]	33 [21; 51]	1.7 [1.2; 2.4]	45 [31; 65]	4.1 [3.4; 5]	105 [85; 132]	<0.001
	Transportation	2.1 [1.6; 2.9]	309 [197; 531]	2.7 [1.9; 3.9]	360 [185; 1,138]	2.8 [1.8; 4.3]	284 [182; 461]	2.2 [1.6; 3.1]	269 [181; 434]	3.7 [3; 4.8]	547 [375; 898]	0.003
	Productivity loss	4.1 [2; 8.6]	762 [510; 1,113]	3.3 [1.1; 9.2]	691 [346; 1,249]	5.3 [2.1; 14.9]	577 [273; 1,072]	9.1 [4.4; 17.7]	992 [624; 1,622]	11.7 [7.6; 17.8]	1,013 [749; 1,383]	0.090
	Global		**12,548 [10,220; 17,188]**		**13,292 [10,138; 18,986]**		**12,215 [9,180; 17,250]**		**14,902 [12,466; 18,135]**		**19,676 [17,393; 22,682]**	<0.001

		0 Canister (*n* = 890)		[1; 2] Canisters (*n* = 354)		[3; 5] Canisters (*n* = 331)		[6; 12] Canisters (*n* = 413)		>12 Canisters (*n* = 583)		
>75	Inpatient stays	2.4 [2.2; 2.7]	11,020 [10,012; 12,134]	2.7 [2.3; 3.2]	12,137 [10,511; 14,105]	2.4 [2.2; 2.8]	10,292 [8,948; 11,751]	3.2 [2.7; 4.9]	13,721 [12,001; 15,748]	3.2 [2.7; 4.3]	12,888 [11,601; 14,573]	0.004
	Medicine, surgery, obstetrics	1.8 [1.7; 1.9]	7,379 [6,777; 8,008]	1.9 [1.7; 2.2]	7,980 [7,050; 9,086]	1.9 [1.7; 2.1]	7,716 [6,764; 8,810]	2.6 [2.1; 4.7]	10,069 [8,976; 11,535]	2.7 [2.2; 3.7]	9,510 [8,632; 10,570]	<0.001
	Respiratory system	0.6 [0.6; 0.7]	2,630 [2,288; 3,034]	0.8 [0.6; 0.9]	3,019 [2,480; 3,739]	0.8 [0.7; 0.9]	3,316 [2,775; 4,117]	1 [0.9; 1.2]	4,506 [3,785; 5,395]	1.1 [1; 1.3]	4,627 [4,052; 5,313]	<0.001
	Asthma-related	0.3 [0.3; 0.4]	1,144 [957; 1,413]	0.4 [0.4; 0.6]	1712 [1,312; 2,304]	0.5 [0.4; 0.6]	1709 [1,340; 2,166]	0.6 [0.5; 0.7]	1990 [1,611; 2,462]	0.7 [0.6; 0.9]	2,521 [2,153; 2,964]	<0.001
	Not related to the respiratory system	1.2 [1; 1.3]	4,748 [4,268; 5,279]	1.2 [1; 1.4]	4,960 [4,272; 5,880]	1 [0.9; 1.3]	4,401 [3,759; 5,164]	1.6 [1.1; 3.1]	5,563 [4,738; 6,581]	1.5 [1.1; 2.7]	4,884 [4,266; 5,639]	0.761
	Consultation	14.8 [13.6; 16.6]	383 [362; 406]	20.6 [17.3; 29]	473 [434; 524]	16.7 [15.1; 19]	473 [438; 513]	17.7 [15.7; 21.3]	471 [437; 513]	18.5 [16.7; 22.1]	490 [461; 527]	<0.001
	General practitioner	14 [13.1; 15.1]	289 [263; 323]	16.9 [15.4; 18.5]	332 [296; 371]	15.9 [14.4; 17.7]	328 [292; 379]	16.4 [14.9; 18.1]	359 [319; 419]	15.9 [14.8; 17.2]	338 [310; 367]	<0.001
	Medical procedures	78 [68.9; 89.7]	1,286 [1,157; 1,457]	91.5 [76.6; 113.8]	1,454 [1,236; 1758]	81.8 [67.2; 102.8]	1,449 [1,194; 1807]	111.7 [86.8; 175.5]	1740 [1,451; 2,154]	103.9 [90.9; 121.7]	1795 [1,579; 2081]	<0.001
	Paramedical procedures	145.8 [138.4; 153.6]	1,283 [1,187; 1,411]	185.2 [174.3; 197.4]	1806 [1,567; 2,255]	186.8 [175.1; 200.1]	1,592 [1,448; 1796]	198 [187.1; 210]	1753 [1,617; 1917]	221.5 [211.6; 232.5]	2,272 [2074; 2,573]	<0.001
	Medications	19.3 [17.7; 21.3]	420 [367; 522]	27.9 [25; 31.9]	677 [536; 1,084]	29.7 [26.7; 33.5]	549 [497; 613]	38.1 [34.7; 41.8]	663 [609; 730]	57.9 [54.2; 61.9]	1,137 [1,012; 1,336]	<0.001
	Respiratory system	10.8 [9.8; 11.9]	1,081 [969; 1,207]	14 [12.4; 15.7]	1,534 [1,319; 1803]	14.2 [12.5; 16.3]	1,346 [1,131; 1,618]	15.3 [13.7; 17.1]	1,557 [1,359; 1780]	18.5 [16.9; 20.2]	1821 [1,618; 2050]	<0.001
	Medical devices	2.5 [2.1; 2.9]	532 [452; 638]	3.1 [2.6; 3.7]	709 [572; 893]	3 [2.4; 3.7]	620 [475; 794]	3.1 [2.5; 3.8]	734 [587; 920]	3.9 [3.4; 4.6]	968 [831; 1,131]	<0.001
	Respiratory assistance devices, home oxygen therapy	1 [0.8; 1.4]	24 [19; 33]	2 [1.6; 2.6]	43 [34; 55]	2.2 [1.7; 2.8]	57 [42; 100]	3.2 [2.7; 3.9]	89 [72; 119]	5.1 [4.5; 5.8]	116 [102; 132]	<0.001
	Transportation	4.5 4.1; 4.9]	439 [398; 489]	4.6 [4.1; 5.2]	500 [420; 644]	4.3 [3.7; 5.1]	437 [373; 550]	5 [4.4; 5.8]	563 [468; 766]	5.2 [4.7; 5.9]	608 [515; 819]	0.109
	Productivity loss	0 [0; 0]	0 [0; 1]	0 [0; 0]	0 [0; 0]	0 [0; 0]	0 [0; 0]	0 [0; 0]	0 [0; 0]	0 [0; 0]	0 [0; 0]	0.756
	Global		**15,781 [14,635; 17,006]**		**18,235 [16,404; 20,436]**		**15,918 [14,341; 17,759]**		**20,164 [18,283; 22,576]**		**20,212 [18,635; 21,872]**	<0.001

The study period was 12 months prior to death. Global costs are indicated in bold.

In patients aged 18–75 years old, total medical costs were statistically higher when patients were dispensed more than 12 canisters (*p* < 0.0001). A difference was observed for medication costs, but also for all other cost components except for productivity loss. In patients aged 75+ years old, total medical costs were statistically higher in patients with a dispensation of six or more canisters (*p* < 0.0001). The cost differences were observed for all cost components except transportation and productivity loss.

### Multivariate analysis of factors associated with highest costs

**Figure 3:**
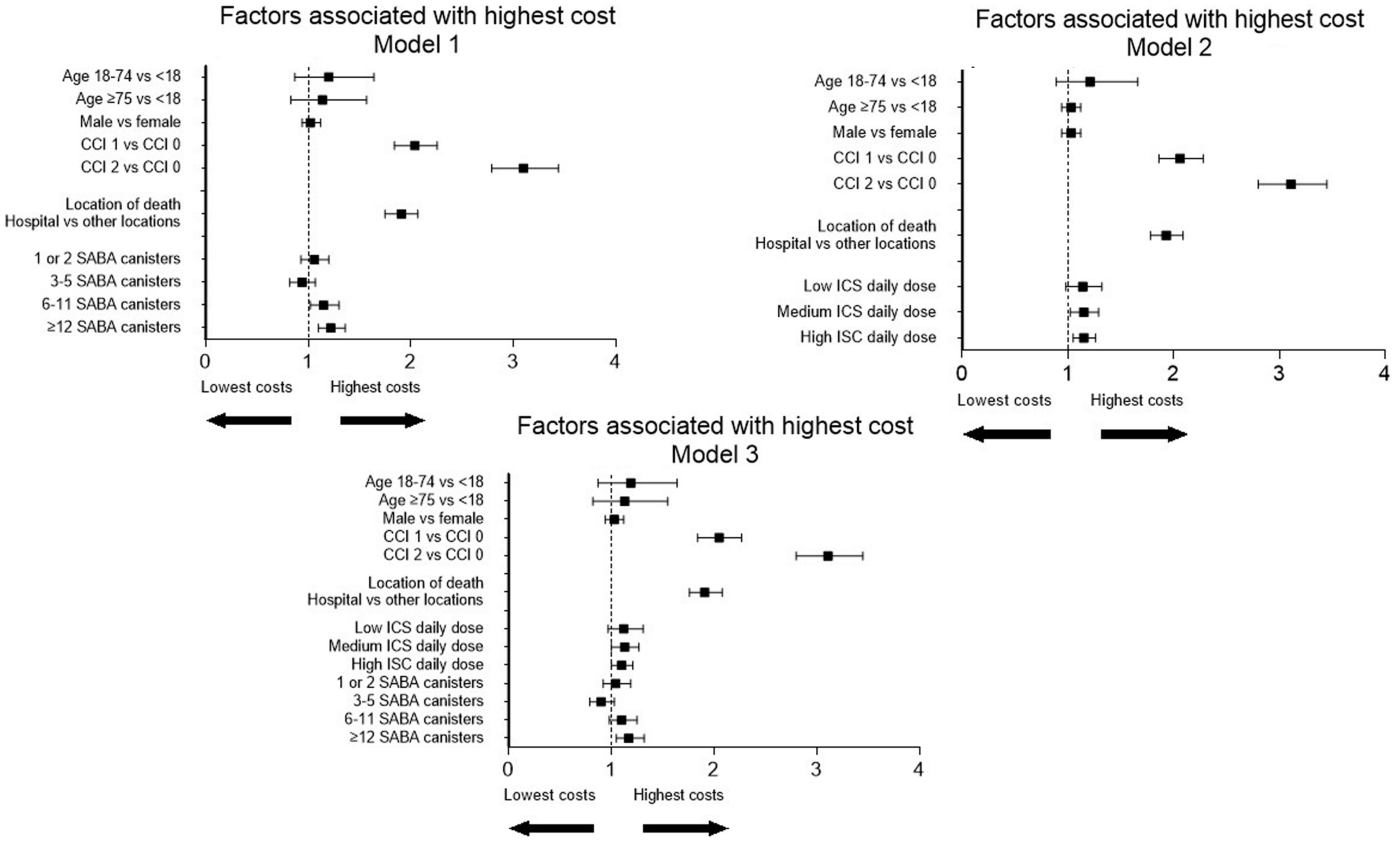
Forest plot of factors associated with the highest costs over 12 months prior to death in patients who died from asthma.

Three models were used for the multivariate analysis of factors associated with the highest costs, depending on the variables included in the regression (Figure 3). On the one hand, age and gender do not influence the cost of patients who died from asthma in all models. On the other hand, comorbidity index ≥1 and death at hospital were associated with the highest costs, regardless of the model. The increase in costs was between 2.04 and 2.06 times higher for a comorbidity index at 1 and between 3.10 and 3.11 times higher for a comorbidity index at 2, according to the three models (*p* < 0.0001 for both comorbidity indices). Costs were 1.91–1.93 times higher with death at hospital compared to other locations of death (*p* < 0.0001). Six or more SABA canisters were associated with the highest costs when ICS dispensation was not included in the analysis. In another model, patients with no ICS had, statistically, the lowest costs compared to patients with moderate or high doses of ICS when SABA dispensation was not included in the analysis. In a model including SABA and ICS dispensation, only 12 or more canisters of SABA were associated with the highest costs compared with no dispensation of SABA canisters (1.17 [1.05; 1.32], *p* = 0.006). Nevertheless, patients with medium and high ICS daily doses and patients with 6–11 SABA canisters tend to be costlier at the threshold boundary.

## Discussion

In patients who died from asthma, total medical costs clearly increased over 24 months prior to death, particularly during the last 6 months prior to death. Over 24 and 12 months prior to death, total medical costs were €27,542 [26,545–28,641] and €16,815 [16,164–17,545], respectively. According to the mortality rate estimated at 0.13% (Supplementary Table S5), the cost per year of asthma death is €16,1 million in France. Costs increased with age, ICS dispensation, and SABA dispensation. According to a multivariate analysis, comorbidity index ≥1, death at hospital, and 12 or more SABA canisters within 12 months prior to death were independently associated with the highest costs (Table 4).

The annual cost of asthma in France ranges from €198 to €4,100, according to asthma severity (21). In Spain, the total annual mean cost of severe asthma rose to €8,554/patient (22). In our study, the total cost within 12 months prior to death in patients who died from asthma was much higher, i.e., €16,815 per patient (2–4 times the cost of severe asthma). Given that two-thirds of the total mean annual costs were due to inpatient stays, the economic burden of patients who died from asthma is mainly due to hospitalization. It has been described that the mean direct cost of severe asthma exacerbations was €758.7/exacerbation, but the mean cost increased by €4,997/exacerbation if only episodes requiring hospital admission were considered (23). It has been shown that an active program for asthma control in Brazil (specialized free medical care, pharmaceutical assistance (inhaled medication), and patient education) leads to a decline in asthma hospital admissions by 82.3% (24). We previously showed that a lack of referral to pulmonologists was observed in patients who died from asthma because less than 15% had a pulmonologist office visit within 12 months prior to death (17). Given that it has been shown that pulmonology office visits appear to reduce the risk of hospitalization for asthma (25), we hypothesize that an increase in referral may have an impact on the cost of asthma death. Indeed, the total cost of the outpatient visit component in our cohort was very low (2.3% of the total cost).

In our study, we show that the economic burden of patients who die from asthma increases with age. A systematic review mentioned that age was found to have an association with asthma costs, but there were conflicting reports (26). According to published data, it seems that extreme ages are associated with excess cost of asthma (27, 28). In the older adult, asthma-derived direct costs are double compared to younger asthma patients, mainly due to the higher costs of hospitalization and medication (29). To the best of our knowledge, there are no data on the impact of age on the economic burden of asthma death. According to the multivariate analysis in our study, age is not associated with the highest costs for patients who died from asthma. However, it seems that comorbidities are associated with the highest costs. Additional costs assessed for older people are related to comorbidity management. Indeed, it has been established that comorbidities account for 54% of the economic burden of severe asthma patients (30). In this study, other respiratory diseases and conditions related to mental, behavioral, neurodevelopmental, digestive, and neurological diseases were the most common comorbidities involved in the economic burden of asthma. Our study highlights the detrimental impact of comorbidities on the economic burden of patients who died from asthma. It is likely that comorbidities are a determining factor in hospitalization. Hospitalization costs and comorbidities are major factors in prolonged hospitalization.

In our study, costs increased with ICS dose prior to asthma death. However, after adjustment, including SABA dispensation, there was no excess of cost regarding the ICS dose. In the models including or not including ICS dose, 12 or more SABA canister dispensation were associated with the highest costs, even for costs not related to medication. It is now well established that over-prescription of SABA is associated with lower odds of controlled asthma and higher severe exacerbation rates, independent of maintenance therapy (31, 32). The overuse of SABA also leads to an excess of mortality (19, 33). In several randomized controlled trials, it has been shown that as-needed ICS-formoterol is more effective than SABA (34–37). It has also been shown that as-needed ICS-formoterol was more effective at preventing severe exacerbations than maintenance low-dose budesonide plus as-needed terbutaline (34). The database used in our study provides data on drug dispensation; however, we have no access to prescription. Consequently, we cannot know if ICS-formoterol was given as-needed or as maintenance therapy. However, our study was conducted before the recommendation of GINA on as-needed ICS-formoterol in mild asthma. For this reason, it is likely that the use of as-needed ICS-Formoterol was not widely used in France during the study period. Moreover, it is worth noticing that the recent French guidelines did not recommend the use of ICS-formoterol as needed in mild asthma (38). ICS-formoterol was only recommended for patients with ICS-formoterol in maintenance therapy. Regarding the economic burden, it has been described that SABA overuse represents greater costs for the Spanish National Health System (39). The impact of SABA dispensation on the economic burden of asthma death has never been described. According to our study, high SABA dispensation is associated with an excess cost, according to several cost components, and mainly for >12 SABA, even in patients who will die from asthma. As suggested in mild asthma, the use of as-needed ICS-formoterol would counterbalance not only the excess risk of death with SABA but also the costs associated with asthma death (40). It has also been described that systemic steroids have a detrimental effect on asthma patients. In our study, we did not include systemic steroids in the analysis because of the risk of bias. Indeed, systemic steroid prescription is not restricted to asthma exacerbations and can be used for a large variety of diseases. Systemic steroid use could reflect comorbidities more than asthma exacerbations.

Our study has limitations. The French health database cannot provide access to biological, functional, or radiological data. For this reason, we are not able to determine asthma phenotypes. This database only offers access to healthcare consumption. However, this database gives the opportunity to make a robust economic analysis of patients who died from asthma with medical costs and costs related to absence in the workplace and who receive a daily allowance from French healthcare insurance and no loss of follow-up in almost the whole French population. Another limitation is the lack of data on drug adherence. We collect data on drug dispensation, but we do not have information regarding drug compliance. Similarly, the prescriptions issued by physicians are not available in this database. Regarding the prescriptions of oral corticosteroids (OCS), the French healthcare database does not offer the possibility of knowing if comorbidities are related to OCS or if OCS were prescribed for asthma exacerbations. Considering the risk of bias, OCS were not included in the analysis. However, it could be interesting to specifically study the role of OCS-related comorbidities in the economic burden of asthma death. Finally, no data on socio-economic status are available in our study, whereas it is well established that the most deprived socio-economic status is more likely to be associated with uncontrolled asthma (41).

## Conclusion

To conclude, the economic burden of asthma death is high and increases with age, ICS dose, and SABA dispensation. Our study also highlights that an increase in healthcare costs over time for asthma patients should alert them to the risk of asthma death. Specific studies on algorithm based on healthcare costs could help in the prediction of asthma death. Moreover, we suggest that prevention costs could be counterbalanced by cost-saving opportunities during the time prior to death from asthma. For example, the cost of referral to a specialist would counterbalance the cost of death and all healthcare resources used for asthma care. This should help the authorities to establish a prevention program according to our results.

In our study, in a multivariate analysis, only hospital as the location of death, comorbidities, and 12 or more SABA canister dispensations were associated with a significant higher cost. The excess cost of SABA over-prescription in asthma death should prompt the authorities to assess if SABA reduction with the use of as-needed ICS-formoterol could lead to decreased asthma death with cost-saving impacts. Moreover, at a clinical level, comorbidities should be taken into account in severe asthma patients’ evaluation. Moreover, the development of outpatient networks that would enable the continued management of asthma exacerbations and comorbidities should be a priority. Finally, the use of biologics should be particularly discussed in patients with comorbidities, given cost savings and death prevention.

## Data availability statement

The raw data supporting the conclusions of this article will be made available by the authors, without undue reservation.

## Ethics statement

The studies involving humans were approved by study protocol by the French National Ethics Committee (registration number TPS 578472). The protocol was also approved by the French Data Protection Authority (registration number 919303v1). The studies were conducted in accordance with the local legislation and institutional requirements. The participants provided their written informed consent to participate in this study.

## Author contributions

LG: conceptualization, formal analysis, funding acquisition, investigation, methodology, project administration, supervision, validation, visualization, writing – original draft, writing – review and editing, data curation resources, and software. MM, AS, CCo, and NC: formal analysis, funding acquisition, methodology, project administration, supervision, validation, visualization, writing – original draft, writing – review and editing, data curation resources, and software. AD, CCa, and LR: methodology, project administration, validation, visualization, writing – original draft, and writing – review and editing. All authors contributed to the article and approved the submitted version.

## References

[1] Global Asthma Network. The Global Asthma Report 2014. Auckland, New Zealand: Global Asthma Network (2014). 769 p.

[2] Jacob C Bechtel B Engel S Kardos P Linder R Braun S. Healthcare costs and resource utilization of asthma in Germany: a claims data analysis. Eur J Health Econ. (2016) 17:195–201. doi: 10.1007/s10198-015-0671-3, PMID: 25716136 PMC4757601

[3] Tavakoli H FitzGerald JM Chen W Lynd L Kendzerska T Aaron S. Ten-year trends in direct costs of asthma: a population-based study. Allergy. (2017) 72:291–9. doi: 10.1111/all.12993, PMID: 27455382

[4] Roche N Nadif R Fabry-Vendrand C Pillot L Thabut G Teissier C. Asthma burden according to treatment steps in the French population-based cohort CONSTANCES. Respir Med. (2022) 206:107057. doi: 10.1016/j.rmed.2022.107057 36502568

[5] Nurmagambetov T Kuwahara R Garbe P. The economic burden of asthma in the United States, 2008-2013. Ann Am Thorac Soc. (2018) 15:348–56. doi: 10.1513/AnnalsATS.201703-259OC, PMID: 29323930

[6] Sullivan PW Slejko JF Ghushchyan VH Sucher B Globe DR Lin SL. The relationship between asthma, asthma control and economic outcomes in the United States. J Asthma. (2014) 51:769–78. doi: 10.3109/02770903.2014.906607 24697738

[7] Zafari Z Sadatsafavi M Chen W FitzGerald JM. The projected economic and health burden of sub-optimal asthma control in Canada. Respir Med. (2018) 138:7–12. doi: 10.1016/j.rmed.2018.03.018, PMID: 29724396

[8] Yaghoubi M Adibi A Safari A FitzGerald JM Sadatsafavi M. The projected economic and health burden of uncontrolled asthma in the United States. Am J Respir Crit Care Med. (2019) 200:1102–12. doi: 10.1164/rccm.201901-0016OC, PMID: 31166782 PMC6888652

[9] Chen S Golam S Myers J Bly C Smolen H Xu X. Systematic literature review of the clinical, humanistic, and economic burden associated with asthma uncontrolled by GINA steps 4 or 5 treatment. Curr Med Res Opin. (2018) 34:2075–88. doi: 10.1080/03007995.2018.1505352 30047292

[10] Settipane RA Kreindler JL Chung Y Tkacz J. Evaluating direct costs and productivity losses of patients with asthma receiving GINA 4/5 therapy in the United States. Ann Allergy Asthma Immunol. (2019) 123:564–72.e3. doi: 10.1016/j.anai.2019.08.462, PMID: 31494235

[11] Chen W Lynd LD FitzGerald JM Marra CA Balshaw R To T. Excess medical costs in patients with asthma and the role of comorbidity. Eur Respir J. (2016) 48:1584–92. doi: 10.1183/13993003.01141-2016, PMID: 27824603

[12] Lefebvre P Duh MS Lafeuille MH Gozalo L Desai U Robitaille MN. Burden of systemic glucocorticoid-related complications in severe asthma. Curr Med Res Opin. (2017) 33:57–65. doi: 10.1080/03007995.2016.1233101, PMID: 27627132

[13] Delmas M Fuhrman C. Asthma in France: a review of descriptive epidemiological data. Rev Mal Respir. (2010) 27:151–9. doi: 10.1016/j.rmr.2009.09.001, PMID: 20206063

[14] Ebmeier S Thayabaran D Braithwaite I Bénamara C Weatherall M Beasley R. Trends in international asthma mortality: analysis of data from the WHO mortality database from 46 countries (1993–2012). Lancet. (2017) 390:935–45. doi: 10.1016/S0140-6736(17)31448-4, PMID: 28797514

[15] Shaw DE Gaynor CM Fogarty AW. Changes in asthma mortality in England and Wales since 2001. Thorax. (2019) 74:1174–5. doi: 10.1136/thoraxjnl-2019-213350, PMID: 31519814

[16] Asthma and Lung UK. (2019). Available at: https://www.asthma.org.uk/about/media/news/press-release-asthma-death-toll-in-england-and-wales-is-the-highest-this-decade/ (Accessed February 8, 2024).

[17] Guilleminault L Mounié M Sommet A Camus C Didier A Reber LL. Healthcare resource consumption prior to asthma-related death: a nationwide descriptive study. Ther Adv Respir Dis. (2022) 16:175346662211302. doi: 10.1177/17534666221130217 PMC9577087 36239261

[18] Bannay A Chaignot C Blotière PO Basson M Weill A Ricordeau P. The best use of the Charlson comorbidity index with electronic health care database to predict mortality. Med Care. (2016) 54:188–94. doi: 10.1097/MLR.0000000000000471, PMID: 26683778

[19] Nwaru BI Ekström M Hasvold P Wiklund F Telg G Janson C. Overuse of short-acting β(2)-agonists in asthma is associated with increased risk of exacerbation and mortality: a nationwide cohort study of the global SABINA programme. Eur Respir J. (2020) 55:1901872. doi: 10.1183/13993003.01872-2019, PMID: 31949111 PMC7160635

[20] Global Initiative for Asthma (GINA). GINA Pocket Guide 2020; (2020). Available at: https://ginasthma.org/wp-content/uploads/2020/04/Main-pocket-guide_2020_04_03-final-wms.pdf (Accessed February 8, 2024).

[21] Gadenne S Pribil C Chouaid C Vergnenegre A Detournay B. The costs of asthma in France and the economic implications of its level of control. Rev Mal Respir. (2011) 28:419–26. doi: 10.1016/j.rmr.2010.11.001 21549898

[22] Melero Moreno C Quirce S Huerta A Uría E Cuesta M. Economic impact of severe asthma in Spain: multicentre observational longitudinal study. J Asthma. (2019) 56:861–71. doi: 10.1080/02770903.2018.1499035 30003827

[23] Quirce S Melero C Huerta A Uría E Cuesta M. Economic impact of severe asthma exacerbations in Spain: multicentre observational study. J Asthma. (2021) 58:207–12. doi: 10.1080/02770903.2019.1674330, PMID: 31621441

[24] Souza-Machado C Souza-Machado A Franco R Ponte EV Barreto ML Rodrigues LC. Rapid reduction in hospitalisations after an intervention to manage severe asthma. Eur Respir J. (2010) 35:515–21. doi: 10.1183/09031936.00101009, PMID: 19643941

[25] Erickson S Tolstykh I Selby JV Mendoza G Iribarren C Eisner MD. The impact of allergy and pulmonary specialist care on emergency asthma utilization in a large managed care organization. Health Serv Res. (2005) 40:1443–65. doi: 10.1111/j.1475-6773.2005.00410.x, PMID: 16174142 PMC1361198

[26] Bahadori K Doyle-Waters MM Marra C Lynd L Alasaly K Swiston J. Economic burden of asthma: a systematic review. BMC Pulm Med. (2009) 9:24. doi: 10.1186/1471-2466-9-24, PMID: 19454036 PMC2698859

[27] Lai C Kim Y Kuo S Spencer M Williams A. Cost of asthma in the Asia-Pacific region. Eur Respir Rev. (2006) 15:10–6. doi: 10.1183/09059180.06.00009802

[28] Stroupe KT Gaskins D Murray MD. Health-care costs of inner-city patients with asthma. J Asthma. (1999) 36:645–55. doi: 10.3109/02770909909055416 10609619

[29] Hoskins G McCowan C Neville RG Thomas GE Smith B Silverman S. Risk factors and costs associated with an asthma attack. Thorax. (2000) 55:19–24. doi: 10.1136/thorax.55.1.19, PMID: 10607797 PMC1745605

[30] Chen W Safari A FitzGerald JM Sin DD Tavakoli H Sadatsafavi M. Economic burden of multimorbidity in patients with severe asthma: a 20-year population-based study. Thorax. (2019) 74:1113–9. doi: 10.1136/thoraxjnl-2019-213223, PMID: 31534029

[31] Bateman ED Price DB Wang HC Khattab A Schonffeldt P Catanzariti A. Short-acting β(2)-agonist prescriptions are associated with poor clinical outcomes of asthma: the multi-country, cross-sectional SABINA III study. Eur Respir J. (2022) 59:2101402. doi: 10.1183/13993003.01402-2021, PMID: 34561293 PMC9068976

[32] Quint JK Arnetorp S Kocks JWH Kupczyk M Nuevo J Plaza V. Short-acting Beta-2-agonist exposure and severe asthma exacerbations: SABINA findings from Europe and North America. J Allergy Clin Immunol Pract. (2022) 10:2297–309.e10. doi: 10.1016/j.jaip.2022.02.047, PMID: 35364341

[33] Tupper OD Ulrik CS. Long-term predictors of severe exacerbations and mortality in a cohort of well-characterised adults with asthma. Respir Res. (2021) 22:269. doi: 10.1186/s12931-021-01864-z, PMID: 34670588 PMC8529759

[34] Hardy J Baggott C Fingleton J Reddel HK Hancox RJ Harwood M. Budesonide-formoterol reliever therapy versus maintenance budesonide plus terbutaline reliever therapy in adults with mild to moderate asthma (PRACTICAL): a 52-week, open-label, multicentre, superiority, randomised controlled trial. Lancet. (2019) 394:919–28. doi: 10.1016/S0140-6736(19)31948-8, PMID: 31451207

[35] O'Byrne PM FitzGerald JM Bateman ED Barnes PJ Zhong N Keen C. Inhaled combined budesonide-formoterol as needed in mild asthma. N Engl J Med. (2018) 378:1865–76. doi: 10.1056/NEJMoa1715274 29768149

[36] Bateman ED Reddel HK O'Byrne PM Barnes PJ Zhong N Keen C. As-needed budesonide-formoterol versus maintenance budesonide in mild asthma. N Engl J Med. (2018) 378:1877–87. doi: 10.1056/NEJMoa1715275, PMID: 29768147

[37] Beasley R Holliday M Reddel HK Braithwaite I Ebmeier S Hancox RJ. Controlled trial of budesonide-formoterol as needed for mild asthma. N Engl J Med. (2019) 380:2020–30. doi: 10.1056/NEJMoa1901963, PMID: 31112386

[38] Raherison-Semjen C Guilleminault L Billiart I Chenivesse C De Oliveira A Izadifar A. Updated guidelines (2021) for management and follow-up of asthmatic patients of the French Society of Pneumology (SPLF) and the French Society of Pediatric Pneumology and Allergology (SP2A). Short version. Respir Med Res. (2022) 81:100898. doi: 10.1016/j.resmer.2022.100898 35526320

[39] Valero A Molina J Nuevo J Simon S Capel M Sicras-Mainar A. Economic consequences of the overuse of short-acting beta-adrenergic agonists (SABA) in the treatment of asthma in Spain. J Investig Allergol Clin Immunol. (2023) 33:109–18. doi: 10.18176/jiaci.0767 34825651

[40] Buendía JA Patiño DG. Cost-utility of as-needed ICS-formoterol versus to maintenance ICS in mild to moderate persistent asthma. BMC Pulm Med. (2021) 21:397. doi: 10.1186/s12890-021-01775-1, PMID: 34865628 PMC8647356

[41] Busby J Price D Al-Lehebi R Bosnic-Anticevich S van Boven JFM Emmanuel B. Impact of socioeconomic status on adult patients with asthma: a population-based cohort Study from UK primary care. J Asthma Allergy. (2021) 14:1375–88. doi: 10.2147/JAA.S326213, PMID: 34785911 PMC8591110

