# Supplementary material for: The economic burden of asthma prior to death: a nationwide descriptive study
Source: Front Public Health. 2024 Feb 19;12:1191788. doi: 10.3389/fpubh.2024.1191788 (PMC10909909; doi:10.3389/fpubh.2024.1191788)
Supplement: Supplementary file 1 [file Table_1.docx]

Supplementary Table 1: Details of medical costs (€2020) in patients who died from asthma over 24 months prior to death. CI : Confident Interval ; Other inpatient care* : Hospital reserved drugs, hospital outpatient department, emergency, dialysis, ** Other consultations: Teleconsultations, mark-up, flat-rate participation; Other medical acts***: anesthesia, surgery, dental, obstetrics ; Other paramedical acts†: pedicure-podiatrist, speech therapists, orthoptists, midwives, thermal baths; Other transportation‡ : personal vehicles, public transport

|  | Period 1: ]-24; -18 ] months | | Period 1: ]-18; -12 ] months | | Period 1: ]-12; -6 ] months | | Period : ]-6; 0] months | | Period All : ]-24; -0] months | |
| --- | --- | --- | --- | --- | --- | --- | --- | --- | --- | --- |
|  | Frequency | Cost | Frequency | Cost | Frequency | Cost | Frequency | Cost | Frequency | Cost |
|  | Mean  [95 CI] | Mean  [95 CI] | Mean  [95 CI] | Mean  [95 CI] | Mean  [95 CI] | Mean  [95 CI] | Mean  [95 CI] | Mean  [95 CI] | Mean  [95 CI] | Mean  [95 CI] |
| **Inpatient stays** | 0.7 [0.6 ; 0.8] | 2698 [2435 ; 3032] | 0.8 [0.7 ; 0.9] | 3179 [2835 ; 3651] | 0.9 [0.8 ; 1.1] | 3561 [3237 ; 3993] | 1.8 [1.6 ; 1.9] | 7708 [7336 ; 8137] | 4.2 [3.9 ; 4.7] | 17146 [16309 ; 18080] |
| ***Medicine, Surgery, Obstetrics*** | 0.6 [0.5 ; 0.7] | 1609 [1481 ; 1758] | 0.6 [0.6 ; 0.8] | 1918 [1747 ; 2208] | 0.7 [0.7 ; 0.9] | 2165 [2001 ; 2356] | 1.4 [1.3 ; 1.6] | 6121 [5817 ; 6461] | 3.4 [3.1 ; 3.8] | 11813 [11278 ; 12387] |
| *Respiratory system* | 0.2 [0.1 ; 0.2] | 552 [485 ; 631] | 0.2 [0.1 ; 0.2] | 663 [563 ; 919] | 0.2 [0.2 ; 0.2] | 804 [710 ; 939] | 0.6 [0.5 ; 0.6] | 2656 [2469 ; 2869] | 1.1 [1 ; 1.2] | 4675 [4367 ; 5065] |
| Asthma related | 0.1 [0.1 ; 0.1] | 314 [269 ; 367] | 0.1 [0.1 ; 0.1] | 362 [310 ; 429] | 0.1 [0.1 ; 0.2] | 466 [400 ; 563] | 0.3 [0.3 ; 0.4] | 1311 [1202 ; 1436] | 0.7 [0.6 ; 0.7] | 2452 [2259 ; 2674] |
| Others | 0.1 [0 ; 0.1] | 238 [196 ; 291] | 0.1 [0 ; 0.1] | 301 [219 ; 599] | 0.1 [0.1 ; 0.1] | 338 [278 ; 429] | 0.2 [0.2 ; 0.2] | 1345 [1208 ; 1513] | 0.4 [0.4 ; 0.4] | 2222 [2024 ; 2523] |
| *Not related to the Respiratory system* | 0.4 [0.3 ; 0.5] | 1057 [954 ; 1177] | 0.5 [0.4 ; 0.6] | 1255 [1132 ; 1445] | 0.5 [0.4 ; 0.7] | 1360 [1241 ; 1494] | 0.9 [0.8 ; 1] | 3466 [3237 ; 3761] | 2.3 [2 ; 2.7] | 7138 [6753 ; 7568] |
| Rehabilitation | 0.1 [0.1 ; 0.2] | 823 [657 ; 1066] | 0.1 [0.1 ; 0.1] | 915 [744 ; 1241] | 0.1 [0.1 ; 0.2] | 1069 [827 ; 1486] | 0.2 [0.2 ; 0.3] | 1243 [1089 ; 1411] | 0.6 [0.6 ; 0.9] | 4049 [3591 ; 4621] |
| Home Care Service | 0 [0 ; 0.1] | 43 [10 ; 164] | 0 [0 ; 0.1] | 120 [32 ; 562] | 0 [0 ; 0.1] | 105 [58 ; 188] | 0.1 [0.1 ; 0.1] | 193 [134 ; 287] | 0.2 [0.1 ; 0.3] | 461 [303 ; 781] |
| Others* | 0 [0 ; 0] | 223 [144 ; 400] | 0 [0 ; 0] | 226 [152 ; 361] | 0 [0 ; 0] | 223 [154 ; 352] | 0 [0 ; 0] | 151 [120 ; 213] | 0 [0 ; 0.1] | 823 [630 ; 1142] |
| Outpatient care service : pneumologist consultation | 0.1 [0 ; 0.1] | 2 [1 ; 2] | 0.1 [0.1 ; 0.1] | 2 [1 ; 2] | 0.1 [0.1 ; 0.1] | 2 [2 ; 3] | 0.1 [0.1 ; 0.1] | 2 [2 ; 2] | 0.3 [0.2 ; 0.3] | 7 [6 ; 9] |
| **Consultations** | 6.7 [6.4 ; 7.1] | 168 [163 ; 174] | 6.8 [6.5 ; 7.2] | 174 [168 ; 180] | 7.2 [6.8 ; 7.7] | 181 [175 ; 187] | 8.3 [7.9 ; 8.9] | 212 [205 ; 218] | 28.9 [27.7 ; 30.3] | 735 [714 ; 756] |
| General practitioner | 5.8 [5.6 ; 6.2] | 124 [119 ; 128] | 6 [5.7 ; 6.4] | 128 [123 ; 133] | 6.3 [6 ; 6.8] | 133 [128 ; 137] | 7.5 [7.1 ; 8.1] | 156 [151 ; 161] | 25.7 [24.5 ; 27.1] | 540 [524 ; 557] |
| Specialist | 0.8 [0.8 ; 0.9] | 23 [21 ; 25] | 0.8 [0.7 ; 0.8] | 22 [20 ; 24] | 0.8 [0.8 ; 1] | 24 [22 ; 27] | 0.8 [0.7 ; 0.9] | 23 [21 ; 25] | 3.2 [3 ; 3.5] | 92 [85 ; 99] |
| Pulmonologist | 0.1 [0.1 ; 0.1] | 2 [1 ; 3] | 0.1 [0 ; 0.1] | 1 [1 ; 2] | 0.1 [0.1 ; 0.1] | 2 [1 ; 2] | 0.1 [0.1 ; 0.1] | 2 [1 ; 2] | 0.3 [0.2 ; 0.3] | 6 [5 ; 8] |
| Other* | 0 [0 ; 0] | 22 [21 ; 23] | 0 [0 ; 0] | 24 [23 ; 26] | 0 [0 ; 0] | 25 [23 ; 26] | 0 [0 ; 0] | 33 [31 ; 35] | 0 [0 ; 0] | 104 [99 ; 109] |
| **Medical procedures** | 6.1 [5.8 ; 6.3] | 145 [137 ; 154] | 6.2 [5.9 ; 6.5] | 144 [137 ; 156] | 6.5 [6.3 ; 6.8] | 154 [145 ; 165] | 6.8 [6.6 ; 7.1] | 158 [151 ; 167] | 25.6 [24.8 ; 26.5] | 601 [576 ; 630] |
| Imaging | 0.6 [0.6 ; 0.6] | 30 [28 ; 33] | 0.6 [0.5 ; 0.6] | 28 [26 ; 30] | 0.6 [0.5 ; 0.6] | 29 [27 ; 32] | 0.6 [0.5 ; 0.6] | 30 [27 ; 32] | 2.3 [2.2 ; 2.4] | 117 [111 ; 124] |
| Laboratory tests | 4.6 [4.3 ; 4.8] | 59 [56 ; 62] | 4.7 [4.5 ; 5] | 61 [58 ; 65] | 5.1 [4.9 ; 5.4] | 67 [64 ; 70] | 5.5 [5.3 ; 5.8] | 75 [71 ; 78] | 20 [19.3 ; 20.7] | 262 [251 ; 274] |
| Others*** | 0.9 [0.8 ; 1] | 56 [51 ; 62] | 0.9 [0.8 ; 1] | 55 [50 ; 64] | 0.8 [0.7 ; 0.9] | 58 [52 ; 66] | 0.7 [0.7 ; 0.9] | 54 [49 ; 60] | 3.3 [3.1 ; 3.8] | 222 [207 ; 240] |
| **Paramedical procedures** | 31.8 [29.1 ; 35.7] | 485 [449 ; 529] | 34.9 [32 ; 39.2] | 544 [504 ; 590] | 37.2 [34.2 ; 41.6] | 575 [534 ; 620] | 37.7 [35 ; 41.2] | 617 [576 ; 665] | 141.6 [131.5 ; 155.5] | 2222 [2076 ; 2397] |
| Nurse | 17.1 [15 ; 19.9] | 239 [210 ; 276] | 17.6 [15.8 ; 20] | 265 [234 ; 306] | 18 [16.3 ; 20.4] | 270 [240 ; 306] | 17.3 [15.6 ; 19.2] | 291 [259 ; 331] | 70 [63.8 ; 78.1] | 1064 [953 ; 1196] |
| Physiotherapist | 14.3 [12.8 ; 16.3] | 205 [190 ; 222] | 16.8 [14.8 ; 20.6] | 232 [215 ; 251] | 18.8 [16.7 ; 22.7] | 258 [239 ; 277] | 20 [17.9 ; 22.6] | 279 [261 ; 299] | 69.9 [63.1 ; 80.3] | 975 [911 ; 1044] |
| Other† | 0.4 [0.3 ; 0.6] | 41 [35 ; 53] | 0.5 [0.4 ; 0.8] | 47 [39 ; 58] | 0.4 [0.3 ; 0.6] | 47 [41 ; 57] | 0.4 [0.3 ; 0.8] | 47 [41 ; 56] | 1.7 [1.3 ; 2.3] | 183 [157 ; 223] |
| **Medications** | 78.4 [76.3 ; 80.6] | 815 [774 ; 867] | 80.6 [78.5 ; 82.8] | 832 [789 ; 892] | 83 [80.8 ; 85.2] | 856 [813 ; 910] | 87.1 [84.9 ; 89.4] | 883 [840 ; 935] | 329.1 [321.2 ; 337] | 3386 [3237 ; 3585] |
| Alimentary tract and metabolism | 10.1 [9.7 ; 10.6] | 70 [65 ; 75] | 10.4 [9.9 ; 10.9] | 69 [64 ; 75] | 10.5 [10 ; 11] | 69 [64 ; 75] | 10.9 [10.5 ; 11.4] | 67 [63 ; 73] | 41.9 [40.2 ; 43.7] | 275 [258 ; 297] |
| Blood and blood forming organs | 3.6 [3.4 ; 3.9] | 43 [38 ; 53] | 4 [3.7 ; 4.2] | 47 [41 ; 54] | 4.2 [4 ; 4.5] | 57 [49 ; 68] | 6 [5.6 ; 6.5] | 69 [62 ; 79] | 17.8 [17 ; 18.8] | 216 [195 ; 244] |
| Cardiovascular system | 8.7 [8.4 ; 9] | 85 [82 ; 89] | 8.9 [8.6 ; 9.2] | 82 [78 ; 86] | 8.9 [8.6 ; 9.3] | 77 [73 ; 80] | 8.7 [8.4 ; 9.1] | 69 [66 ; 72] | 35.3 [34.1 ; 36.5] | 312 [300 ; 326] |
| Dermatologicals | 1.8 [1.6 ; 2] | 5 [4 ; 6] | 1.7 [1.6 ; 1.9] | 5 [4 ; 5] | 1.8 [1.7 ; 2.1] | 4 [3 ; 5] | 1.8 [1.7 ; 2.1] | 4 [4 ; 5] | 7.2 [6.6 ; 7.9] | 17 [16 ; 20] |
| Genito-urinary system and sex hormones | 0.7 [0.6 ; 0.8] | 4 [4 ; 5] | 0.6 [0.6 ; 0.8] | 5 [4 ; 5] | 0.7 [0.6 ; 1] | 5 [4 ; 5] | 0.7 [0.6 ; 1.1] | 5 [5 ; 7] | 2.7 [2.4 ; 3.6] | 19 [17 ; 22] |
| Systemic hormonal preparations | 2.9 [2.7 ; 3.1] | 12 [10 ; 15] | 3 [2.8 ; 3.3] | 11 [9 ; 15] | 3.2 [3 ; 3.5] | 13 [10 ; 16] | 3.7 [3.5 ; 4] | 13 [11 ; 17] | 12.9 [12.1 ; 13.8] | 49 [42 ; 60] |
| Antiinfectives for systemic use | 3.7 [3.5 ; 4] | 38 [33 ; 49] | 4.1 [3.8 ; 4.3] | 44 [38 ; 56] | 4.3 [4 ; 4.6] | 47 [40 ; 59] | 5.5 [5.3 ; 5.9] | 55 [48 ; 66] | 17.6 [16.8 ; 18.5] | 185 [161 ; 228] |
| Antineoplastic and immunomodulating agents | 0.2 [0.2 ; 0.3] | 42 [25 ; 84] | 0.2 [0.2 ; 0.3] | 45 [26 ; 105] | 0.2 [0.2 ; 0.2] | 42 [26 ; 85] | 0.2 [0.2 ; 0.3] | 53 [35 ; 83] | 0.8 [0.7 ; 1] | 183 [120 ; 338] |
| Musculo-skeletal system | 1.9 [1.8 ; 2] | 13 [11 ; 16] | 1.7 [1.6 ; 1.8] | 12 [10 ; 15] | 1.7 [1.5 ; 1.8] | 12 [10 ; 16] | 1.5 [1.4 ; 1.7] | 11 [9 ; 14] | 6.8 [6.4 ; 7.3] | 49 [41 ; 60] |
| Nervous system | 26.9 [25.8 ; 28] | 104 [96 ; 113] | 27.4 [26.4 ; 28.6] | 104 [96 ; 115] | 28.2 [27.2 ; 29.4] | 104 [96 ; 115] | 28.2 [27.1 ; 29.4] | 103 [95 ; 113] | 110.8 [106.8 ; 114.9] | 415 [385 ; 453] |
| Antiparasitic products, insecticides and repellents | 0 [0 ; 0.1] | 0 [0 ; 0] | 0 [0 ; 0.1] | 0 [0 ; 0] | 0.1 [0 ; 0.1] | 1 [0 ; 2] | 0 [0 ; 0.1] | 2 [0 ; 9] | 0.2 [0.2 ; 0.2] | 3 [1 ; 11] |
| **Respiratory system** | 15.7 [15.1 ; 16.3] | 349 [322 ; 382] | 16.3 [15.7 ; 17] | 346 [322 ; 376] | 17 [16.4 ; 17.7] | 358 [332 ; 391] | 17.6 [16.9 ; 18.3] | 359 [334 ; 394] | 66.6 [64.3 ; 69] | 1411 [1320 ; 1528] |
| Sensory organs | 1.9 [1.8 ; 2.1] | 23 [18 ; 31] | 1.9 [1.8 ; 2.1] | 27 [20 ; 38] | 1.8 [1.7 ; 2] | 25 [19 ; 34] | 1.7 [1.6 ; 1.9] | 18 [14 ; 23] | 7.4 [6.9 ; 8] | 93 [75 ; 116] |
| Various | 0.1 [0.1 ; 0.1] | 9 [4 ; 30] | 0.1 [0.1 ; 0.1] | 9 [4 ; 33] | 0.1 [0.1 ; 0.1] | 9 [4 ; 27] | 0.1 [0.1 ; 0.1] | 9 [4 ; 28] | 0.4 [0.3 ; 0.5] | 36 [16 ; 129] |
| Medical deductible | 0.1 [0.1 ; 0.1] | 2 [1 ; 3] | 0.1 [0.1 ; 0.1] | 2 [2 ; 3] | 0.1 [0.1 ; 0.1] | 2 [2 ; 3] | 0.1 [0.1 ; 0.1] | 2 [1 ; 3] | 0.3 [0.3 ; 0.4] | 8 [6 ; 12] |
| Dispensing fee | 0 [0 ; 0] | 16 [15 ; 17] | 0 [0 ; 0] | 23 [22 ; 24] | 0 [0 ; 0] | 32 [31 ; 34] | 0 [0 ; 0] | 42 [40 ; 44] | 0 [0 ; 0] | 113 [108 ; 119] |
| **Medical Device** | 4.6 [4.4 ; 4.9] | 453 [419 ; 489] | 5 [4.8 ; 5.3] | 496 [464 ; 536] | 5.5 [5.2 ; 5.7] | 550 [517 ; 586] | 6.6 [6.3 ; 6.9] | 640 [608 ; 678] | 21.7 [20.8 ; 22.7] | 2139 [2018 ; 2274] |
| Respiratory assistance devices, home oxygen therapy | 1 [0.9 ; 1] | 234 [211 ; 259] | 1.1 [1 ; 1.2] | 254 [232 ; 280] | 1.2 [1.1 ; 1.3] | 288 [264 ; 315] | 1.4 [1.3 ; 1.5] | 299 [276 ; 322] | 4.6 [4.2 ; 5] | 1074 [989 ; 1171] |
| Aerosol generator device | 1 [0.9 ; 1.1] | 23 [21 ; 25] | 1 [0.9 ; 1.1] | 25 [23 ; 27] | 1.1 [1 ; 1.2] | 27 [25 ; 30] | 1.3 [1.2 ; 1.4] | 31 [28 ; 34] | 4.4 [4.1 ; 4.8] | 106 [97 ; 115] |
| Others | 2.7 [2.6 ; 2.9] | 197 [179 ; 223] | 3 [2.8 ; 3.1] | 217 [199 ; 246] | 3.1 [3 ; 3.3] | 235 [215 ; 256] | 3.9 [3.7 ; 4.1] | 311 [289 ; 334] | 12.7 [12.1 ; 13.3] | 959 [897 ; 1038] |
| **Transportation** | 1.4 [1.3 ; 1.5] | 167 [147 ; 193] | 1.5 [1.4 ; 1.7] | 179 [160 ; 207] | 1.7 [1.6 ; 1.8] | 195 [176 ; 221] | 2.4 [2.2 ; 2.5] | 263 [243 ; 291] | 6.9 [6.6 ; 7.3] | 804 [737 ; 897] |
| Ambulance | 0.7 [0.7 ; 0.8] | 96 [84 ; 119] | 0.9 [0.8 ; 1] | 110 [97 ; 132] | 1 [0.9 ; 1.1] | 121 [110 ; 141] | 1.7 [1.6 ; 1.8] | 194 [178 ; 217] | 4.4 [4.1 ; 4.6] | 521 [476 ; 601] |
| light medical vehicles | 0.3 [0.2 ; 0.3] | 18 [14 ; 26] | 0.3 [0.3 ; 0.4] | 19 [15 ; 26] | 0.3 [0.3 ; 0.4] | 21 [16 ; 28] | 0.3 [0.3 ; 0.3] | 17 [14 ; 21] | 1.2 [1.1 ; 1.4] | 74 [61 ; 100] |
| Taxis | 0.3 [0.3 ; 0.4] | 39 [29 ; 58] | 0.3 [0.3 ; 0.4] | 38 [29 ; 53] | 0.3 [0.3 ; 0.4] | 37 [28 ; 54] | 0.3 [0.3 ; 0.4] | 40 [30 ; 55] | 1.2 [1.1 ; 1.4] | 154 [120 ; 218] |
| Other‡ | 0 [0 ; 0] | 13 [9 ; 23] | 0 [0 ; 0] | 12 [9 ; 19] | 0 [0 ; 0.1] | 16 [12 ; 23] | 0 [0 ; 0] | 13 [10 ; 16] | 0.1 [0.1 ; 0.2] | 55 [46 ; 67] |
| **Productivity loss** | 1.1 [0.8 ; 1.6] | 123 [100 ; 150] | 1.1 [0.8 ; 1.5] | 124 [100 ; 151] | 1.1 [0.8 ; 1.6] | 134 [108 ; 169] | 1.1 [0.8 ; 1.5] | 128 [105 ; 155] | 4.5 [3.4 ; 5.8] | 509 [421 ; 611] |
| Dailly allowance | 1.1 [0.8 ; 1.6] | 34 [23 ; 49] | 1.1 [0.8 ; 1.5] | 36 [25 ; 50] | 1.1 [0.8 ; 1.6] | 35 [25 ; 48] | 1.1 [0.8 ; 1.5] | 36 [26 ; 50] | 4.5 [3.5 ; 5.9] | 140 [108 ; 181] |
| Invalidity pension | 0 [0 ; 0] | 89 [71 ; 113] | 0 [0 ; 0] | 88 [69 ; 112] | 0 [0 ; 0] | 99 [76 ; 136] | 0 [0 ; 0] | 92 [73 ; 118] | 0 [0 ; 0] | 369 [294 ; 466] |
| **Global** |  | 5055 [4751 ; 5402] |  | 5673 [5308 ; 6153] |  | 6206 [5834 ; 6666] |  | 10609 [10,184 ; 11,040] |  | 27542 [26,545 ; 28,641] |

Supplementary Table 2: Details of medical costs (€2020) according to age in patients who died from asthma over 12 months prior to death. CI : Confident Interval ; Other inpatient care* : Hospital reserved drugs, hospital outpatient department, emergency, dialysis.. , ** Other consultations: Teleconsultations, mark-up, flat-rate participation; Other medical acts***: anesthesia, surgery, dental, obstetrics ; Other paramedical acts†: pedicure-podiatrist, speech therapists, orthoptists, midwives, thermal baths; Other transportation‡ : personal vehicles, public transport

|  | [0 ; 18[ years (N=64) | | [18; 75[ years (N=1194) | | [75; +] years (N=2571) | |  |
| --- | --- | --- | --- | --- | --- | --- | --- |
|  | Frequency | Cost | Frequency | Cost | Frequency | Cost | P-value |
|  | Mean  [95 CI] | Mean  [95 CI] | Mean  [95 CI] | Mean  [95 CI] | Mean  [95 CI] | Mean  [95 CI] |  |
| **Inpatient stays** |  | **7480 [5438 ; 10883]** |  | **10035 [8886 ; 11435]** |  | **11938 [11320 ; 12645]** | <0.0001 |
| Medicine, Surgery, Obstetrics | 1.8 [1.3 ; 2.8] | **7277 [5268 ; 10710]** | 2.2 [1.8 ; 2.9] | **8050 [7259 ; 9104]** | 2.2 [2 ; 2.5] | **8420 [8021 ; 8862]** | <0.0001 |
| *Respiratory system* | 0.9 [0.6 ; 1.2] | **3660 [2432 ; 5341]** | 0.6 [0.5 ; 0.7] | **3306 [2811 ; 3987]** | 0.9 [0.8 ; 0.9] | **3526 [3279 ; 3790]** | <0.0001 |
| Asthma related | 0.6 [0.5 ; 0.9] | **2393 [1459 ; 4031]** | 0.4 [0.4 ; 0.5] | **1816 [1519 ; 2275]** | 0.5 [0.5 ; 0.5] | **1743 [1588 ; 1908]** | <0.0001 |
| Others | 0.2 [0.1 ; 0.3] | 1267 [615 ; 2493] | 0.2 [0.1 ; 0.2] | 1490 [1171 ; 1946] | 0.4 [0.3 ; 0.4] | 1783 [1609 ; 2001] | 0.0172 |
| *Not related with the Respiratory system* | 1 [0.6 ; 2.2] | 3618 [2142 ; 6677] | 1.6 [1.2 ; 2.3] | 4745 [4152 ; 5606] | 1.3 [1.2 ; 1.6] | 4894 [4604 ; 5241] | 0.0314 |
| Rehabilitation | 0 [0 ; 0] | 35 [0 ; 105] | 0.3 [0.1 ; 0.7] | 1099 [694 ; 2600] | 0.5 [0.4 ; 0.5] | **2931 [2587 ; 3398]** | 0.9419 |
| Home Care Service | 0 [0 ; 0] | 0 [0 ; 0] | 0.1 [0.1 ; 0.2] | 172 [77 ; 410] | 0.1 [0.1 ; 0.2] | 364 [243 ; 547] | 0.2961 |
| Others* | 0 [0 ; 0] | 168 [112 ; 264] | 0 [0 ; 0.1] | 712 [472 ; 1292] | 0 [0 ; 0] | 223 [173 ; 324] | <0.0001 |
| Outpatient pneumologist consultation | 0 [0 ; 0.1] | 2 [0 ; 5] | 0.2 [0.1 ; 0.3] | 5 [4 ; 7] | 0.1 [0 ; 0] | 4 [1 ; 7] | <0.0001 |
| **Consultation** | 7.3 [5.8 ; 9.9] | 140 [114 ; 170] | 12.2 [11.4 ; 13.3] | 292 [274 ; 314] | 17.1 [16.2 ; 18.5] | 445 [432 ; 461] | <0.0001 |
| General practitioner | 6.3 [4.9 ; 9.2] | 104 [83 ; 130] | 10.4 [9.6 ; 11.6] | 204 [192 ; 219] | 15.6 [14.6 ; 16.9] | 332 [320 ; 344] | <0.0001 |
| Specialist | 1 [0.7 ; 1.6] | 27 [18 ; 41] | 1.8 [1.6 ; 2.2] | 55 [47 ; 66] | 1.6 [1.4 ; 1.8] | 44 [40 ; 49] | 0.0081 |
| Pulmonologist | 0  [0 ; 0.1] | 1  [0 ; 2] | 0.1  [0.1 ; 0.2] | 3  [2 ; 4] | 0.1  [0 ; 0] | 4  [2 ; 7] | <0.0001 |
| Others** | 0 [0 ; 0] | 9 [5 ; 15] | 0 [0 ; 0] | 33 [30 ; 38] | 0 [0 ; 0] | 70 [66 ; 73] | 0.0138 |
| **Medical procedures** | 1.5 [1 ; 2.2] | 74 [47 ; 112] | 9.4 [8.7 ; 10.4] | 303 [272 ; 343] | 15.5 [14.9 ; 16.1] | 322 [306 ; 340] | <0.0001 |
| Imaging | 0.4 [0.2 ; 0.8] | 18 [10 ; 36] | 1.4 [1.2 ; 1.5] | 71 [63 ; 81] | 1.1 [1 ; 1.1] | 54 [51 ; 58] | 0.043 |
| Laboratory tests | 0.4 [0.2 ; 0.6] | 15 [9 ; 28] | 6.1 [5.6 ; 6.7] | 95 [85 ; 107] | 13 [12.5 ; 13.6] | 166 [159 ; 175] | 0.0021 |
| Others*** | 0.7 [0.4 ; 1.1] | 40 [23 ; 73] | 2 [1.6 ; 2.7] | 137 [119 ; 165] | 1.4 [1.3 ; 1.5] | 101 [93 ; 114] | <0.0001 |
| **Paramedical procedures** | 7.2 [3.6 ; 16.3] | 125 [66 ; 256] | 42.6 [34.3 ; 53.9] | 547 [451 ; 706] | 91.6 [85 ; 102] | 1518 [1419 ; 1631] | 0.0074 |
| Nurse | 0.5 [0.1 ; 2.1] | 8 [0 ; 38] | 24.7 [19.9 ; 31.8] | 328 [252 ; 475] | 41.1 [37 ; 46.1] | 682 [603 ; 769] | 0.0001 |
| Physiotherapist | 5.5 [2.3 ; 14.8] | 89 [42 ; 226] | 17.5 [12.8 ; 25.7] | 187 [154 ; 231] | 49.5 [44.6 ; 58.5] | 711 [660 ; 761] | 0.2226 |
| Others† | 1.2 [0.3 ; 3] | 27 [8 ; 79] | 0.4 [0.3 ; 0.7] | 32 [20 ; 49] | 1 [0.7 ; 1.5] | 125 [106 ; 150] | 0.802 |
| **Medications** | 43.8 [35.6 ; 54.3] | 358 [272 ; 496] | 151 [143.1 ; 160] | 1906 [1722 ; 2167] | 182.1 [177.6 ; 186.8] | 1695 [1613 ; 1793] | <0.0001 |
| Alimentary tract and metabolism | 2.9 [1.8 ; 4.9] | 8 [3 ; 19] | 17 [15.4 ; 18.9] | 150 [129 ; 178] | 23.9 [22.9 ; 24.9] | 133 [124 ; 144] | <0.0001 |
| Blood and blood forming organs | 1.1 [0.4 ; 3.1] | 6 [1 ; 34] | 4.2 [3.7 ; 4.8] | 69 [54 ; 101] | 13.3 [12.5 ; 14.3] | 155 [138 ; 183] | 0.1423 |
| Cardiovascular system | 0.2 [0 ; 0.5] | 3 [0 ; 13] | 10.5 [9.6 ; 11.5] | 119 [106 ; 133] | 21.4 [20.7 ; 22.2] | 161 [154 ; 169] | 0.0005 |
| Dermatologicals | 2 [1.2 ; 3.4] | 2 [1 ; 4] | 2.9 [2.5 ; 3.5] | 7 [6 ; 9] | 4.1 [3.7 ; 4.8] | 9 [8 ; 10] | 0.0004 |
| Genito-urinary system and sex hormones | 0.2 [0.1 ; 0.5] | 1 [0 ; 2] | 1.5 [0.9 ; 3.7] | 9 [6 ; 14] | 1.4 [1.3 ; 1.6] | 11 [9 ; 13] | 0.78 |
| Systemic hormonal preparations | 2.6 [2 ; 3.3] | 8 [6 ; 15] | 6.4 [5.7 ; 7.8] | 31 [22 ; 48] | 7.2 [6.7 ; 7.8] | 24 [20 ; 30] | <0.0001 |
| Antiinfectives for systemic use | 6 [4.3 ; 9.3] | 39 [25 ; 79] | 8.1 [7.4 ; 8.9] | 129 [93 ; 198] | 10.7 [10.1 ; 11.5] | 92 [81 ; 110] | <0.0001 |
| Antineoplastic and immunomodulating agents | 0 [0 ; 0.1] | 12 [0 ; 36] | 0.3 [0.2 ; 0.5] | 108 [47 ; 278] | 0.4 [0.4 ; 0.6] | 91 [57 ; 156] | 0.7846 |
| Musculo-skeletal system | 0.8 [0.5 ; 1.1] | 1 [1 ; 2] | 3.5 [3.2 ; 4.1] | 19 [14 ; 32] | 3.1 [2.8 ; 3.4] | 26 [21 ; 35] | <0.0001 |
| Nervous system | 6.4 [4.5 ; 10.4] | 26 [6 ; 123] | 56.3 [51.8 ; 61.1] | 295 [253 ; 352] | 57.7 [55.5 ; 60.1] | 170 [159 ; 182] | <0.0001 |
| Antiparasitic products, insecticides and repellents | 0.2 [0 ; 0.5] | 1 [0 ; 2] | 0.1 [0.1 ; 0.2] | 6 [1 ; 38] | 0.1 [0.1 ; 0.1] | 1 [0 ; 6] | 0.1311 |
| **Respiratory system** | 20.5 [16.1 ; 28.1] | 212 [157 ; 327] | 37.4 [35.1 ; 40.2] | 837 [717 ; 1008] | 33.6 [32.2 ; 35.2] | 673 [627 ; 743] | <0.0001 |
| Sensory organs | 0.7 [0.3 ; 1.2] | 1 [0 ; 1] | 2 [1.8 ; 2.4] | 20 [12 ; 38] | 4.4 [4 ; 4.7] | 54 [42 ; 70] | 0.1735 |
| Various | 0 [0 ; 0] | 0 [0 ; 0] | 0.3 [0.2 ; 0.4] | 38 [10 ; 167] | 0.2 [0.1 ; 0.2] | 8 [6 ; 11] | 0.7173 |
| Medical deductible | 0 [0 ; 0.1] | 22 [0 ; 106] | 0.2 [0.1 ; 0.2] | 4 [3 ; 7] | 0.2 [0.1 ; 0.2] | 4 [3 ; 6] | 0.2714 |
| Dispensing honorary | 0 [0 ; 0] | 16 [11 ; 23] | 0 [0 ; 0] | 65 [59 ; 72] | 0 [0 ; 0] | 80 [76 ; 84] | 0.0003 |
| **Medical Device** | 2.9 [1.7 ; 6.4] | 271 [102 ; 850] | 7.9 [7.2 ; 8.8] | 740 [641 ; 847] | 14.2 [13.5 ; 14.9] | 1422 [1340 ; 1515] | <0.0001 |
| Respiratory assistance devices, home oxygen therapy | 0.4 [0.2 ; 0.7] | 22 [4 ; 84] | 1.7 [1.4 ; 2] | 375 [308 ; 452] | 3.1 [2.8 ; 3.3] | 699 [640 ; 764] | <0.0001 |
| Aerosol generator device | 0.7 [0.3 ; 1.7] | 18 [6 ; 43] | 2.1 [1.8 ; 2.5] | 51 [44 ; 61] | 2.6 [2.4 ; 2.9] | 62 [56 ; 70] | <0.0001 |
| Others | 1.7 [0.7 ; 5.7] | 231 [61 ; 851] | 4.1 [3.7 ; 4.7] | 314 [269 ; 384] | 8.5 [8 ; 9] | 660 [614 ; 712] | 0.0065 |
| **Transportation** | 1.4 [0.4 ; 5.5] | 144 [55 ; 327] | 2.7 [2.4 ; 3.2] | 374 [296 ; 491] | 4.7 [4.5 ; 5] | 505 [468 ; 559] | 0.1613 |
| Ambulance | 0.4 [0.1 ; 1.2] | 65 [15 ; 198] | 1.1 [0.9 ; 1.3] | 160 [122 ; 245] | 3.6 [3.4 ; 3.8] | 393 [363 ; 438] | 0.1885 |
| light medical vehicles | 0 [0 ; 0] | 1 [0 ; 4] | 0.7 [0.6 ; 0.9] | 55 [40 ; 83] | 0.6 [0.5 ; 0.7] | 30 [24 ; 42] | 0.0361 |
| Taxis | 1 [0 ; 5.9] | 62 [12 ; 235] | 0.8 [0.7 ; 1.1] | 142 [93 ; 245] | 0.5 [0.5 ; 0.6] | 47 [37 ; 70] | 0.0804 |
| Others‡ | 0.1 [0 ; 0.2] | 15 [2 ; 56] | 0.1 [0.1 ; 0.2] | 16 [10 ; 25] | 0.1 [0.1 ; 0.1] | 35 [28 ; 45] | 0.6123 |
| **Productivity loss** | 0 [0 ; 0] | 0 [0 ; 0] | 7.1 [5.3 ; 9.6] | 840 [688 ; 1007] | 0 [0 ; 0] | 0 [0 ; 0] | 0.0033 |
| Dailly allowance | 0 [0 ; 0] | 0 [0 ; 0] | 7.1 [5.3 ; 9.8] | 226 [170 ; 304] | 0 [0 ; 0] | 0 [0 ; 0] | 0.0034 |
| Invalidity pension | 0 [0 ; 0] | 0 [0 ; 0] | 0 [0 ; 0] | 614 [483 ; 785] | 0 [0 ; 0] | 0 [0 ; 0] | 0.0874 |
| **Global** |  | **8592 [6457 ; 12294]** |  | **15038 [13745 ; 16672]** |  | **17845 [17139 ; 18618]** | **<0.0001** |

Supplementary Table 3: Details of medical costs (€2020) according to inhaled corticosteroid (ICS) dispensation in patients who died from asthma over 12 months prior to death. ICS and ICS/LABA dispensation are expressed in budesonide equivalents. a : ICS daily dose <=100 µg/day for children (<12 years) and <=200 µg/day for other age categories. b: ICS daily dose ]100 ; 200] µg/day for children (<12 years) and ]200 ; 400] µg/day for other age categories. c: ICS daily dose ]200 ; 400] µg/day for children (<12 years) and ]400 ; 800] µg/day for other age categories. d: ICS daily dose >400 µg/day for children (<12 years) and >800 µg/day for other age categories. CI : Confident Interval ; Other inpatient care* : Hospital reserved drugs, hospital outpatient department, emergency, dialysis.. , ** Other consultations: Teleconsultations, mark-up, flat-rate participation; Other medical acts***: anesthesia, surgery, dental, obstetrics … ; Other paramedical acts†: pedicure-podiatrist, speech therapists, orthoptists, midwives, thermal baths ...; Other transportation‡ : personal vehicles, public transport…

|  | 0^a^ | | Low ^b^ | | Moderate ^c^ | | High ^d^ | |  |
| --- | --- | --- | --- | --- | --- | --- | --- | --- | --- |
|  | Frequency | Cost | Frequency | Cost | Frequency | Cost | Frequency | Cost | P-value |
|  | Mean [95 IC] | Mean [95 IC] | Mean [95 IC] | Mean [95 IC] | Mean [95 IC] | Mean [95 IC] | Mean [95 IC] | Mean [95 IC] |  |
| **Inpatient stays** |  | **10394 [9587 ; 11418]** |  | **12447 [10324 ; 14968]** |  | **12561 [11174 ; 14188]** |  | **11642 [10732 ; 12662]** | <0.0001 |
| Medicine, Surgery, Obstetrics | 2 [1.8 ; 2.5] | **7484 [6940 ; 8147]** | 1.8 [1.6 ; 2.1] | **8344 [7202 ; 10019]** | 2.3 [2 ; 3.1] | **9305 [8332 ; 10488]** | 2.4 [2.1 ; 3.1] | **8988 [8386 ; 9725]** | <0.0001 |
| *Respiratory system* | 0.6 [0.6 ; 0.7] | **2743 [2454 ; 3166]** | 0.8 [0.7 ; 1] | **3821 [3058 ; 5377]** | 0.9 [0.8 ; 1.1] | **4243 [3560 ; 5020]** | 0.9 [0.8 ; 1] | **4060 [3634 ; 4607]** | <0.0001 |
| Asthma related | 0.4 [0.3 ; 0.4] | **1434 [1251 ; 1737]** | 0.5 [0.4 ; 0.6] | **1842 [1461 ; 2354]** | 0.6 [0.5 ; 0.8] | **2163 [1799 ; 2614]** | 0.6 [0.5 ; 0.6] | **2088 [1824 ; 2394]** | <0.0001 |
| Others | 0.3 [0.2 ; 0.3] | 1309 [1141 ; 1522] | 0.3 [0.3 ; 0.4] | 1979 [1370 ; 3251] | 0.3 [0.3 ; 0.4] | 2080 [1600 ; 2759] | 0.4 [0.3 ; 0.4] | 1972 [1670 ; 2319] | 0.0001 |
| *Not related with the Respiratory system* | 1.4 [1.1 ; 1.8] | 4741 [4307 ; 5335] | 1 [0.9 ; 1.2] | 4523 [3736 ; 5441] | 1.3 [1.1 ; 2.3] | 5062 [4386 ; 6072] | 1.5 [1.2 ; 2.1] | 4928 [4474 ; 5496] | 0.2414 |
| Rehabilitation | 0.4 [0.3 ; 0.6] | 2365 [1900 ; 3184] | 0.4 [0.3 ; 0.6] | 3452 [2156 ; 5403] | 0.4 [0.3 ; 0.5] | 2176 [1693 ; 2897] | 0.4 [0.3 ; 0.5] | 1978 [1604 ; 2395] | 0.3297 |
| Home Care Service | 0.1 [0.1 ; 0.2] | 216 [123 ; 443] | 0.2 [0.1 ; 0.4] | 426 [124 ; 1223] | 0.1 [0 ; 0.2] | 252 [113 ; 547] | 0.2 [0.1 ; 0.3] | 406 [219 ; 722] | 0.6971 |
| Others* |  | 329 [238 ; 501] |  | 225 [138 ; 596] |  | 828 [400 ; 2117] |  | 270 [186 ; 458] | 0.2811 |
| Outpatient pulmonologist consultation | 0.1 [0.1 ; 0.1] | 3 [2 ; 4] | 0.1 [0.1 ; 0.2] | 3 [2 ; 5] | 0.2 [0.1 ; 0.3] | 5 [4 ; 8] | 0.2 [0.1 ; 0.2] | 5 [4 ; 7] | <0.0001 |
| **Consultation** | 14.2 [13.2 ; 15.9] | **352 [336 ; 370]** | 14.8 [12.8 ; 19.3] | **367 [333 ; 409]** | 18.1 [16.2 ; 21.8] | **436 [406 ; 471]** | 16.3 [15.3 ; 17.4] | **440 [418 ; 462]** | <0.0001 |
| General practitioner | 12.7 [11.6 ; 14.4] | **258 [246 ; 272]** | 13.5 [11.4 ; 18.5] | **278 [251 ; 316]** | 16.1 [14.5 ; 19.6] | **320 [296 ; 348]** | 14.5 [13.5 ; 15.6] | **322 [306 ; 339]** | <0.0001 |
| Specialist | 1.5 [1.3 ; 1.7] | 42 [37 ; 50] | 1.3 [1.1 ; 1.7] | 38 [30 ; 50] | 2.1 [1.7 ; 2.7] | 58 [49 ; 70] | 1.8 [1.5 ; 2.3] | 51 [45 ; 62] | <0.0001 |
| Pulmonologist | 0.1 [0.1 ; 0.1] | 2 [2 ; 3] | 0.1 [0.1 ; 0.2] | 3 [2 ; 5] | 0.1 [0.1 ; 0.2] | 4 [2 ; 5] | 0.2 [0.1 ; 0.2] | 5 [4 ; 6] | <0.0001 |
| Others** | 0 [0 ; 0] | 52 [48 ; 56] | 0 [0 ; 0] | 50 [43 ; 62] | 0 [0 ; 0] | 58 [52 ; 66] | 0 [0 ; 0] | 67 [62 ; 73] | <0.0001 |
| **Medical procedures** | **12.3 [11.6 ; 13]** | **277 [258 ; 301]** | **11.9 [10.5 ; 13.3]** | **281 [242 ; 352]** | **14.8 [13.6 ; 16.1]** | **345 [310 ; 395]** | **14.7 [13.8 ; 15.6]** | **358 [331 ; 390]** | <0.0001 |
| Imaging | 1 [0.9 ; 1.1] | 52 [47 ; 58] | 1 [0.9 ; 1.2] | 48 [39 ; 58] | 1.4 [1.2 ; 1.6] | 72 [62 ; 84] | 1.2 [1.1 ; 1.3] | 66 [59 ; 75] | 0.0001 |
| Laboratory tests | 9.8 [9.2 ; 10.4] | 127 [119 ; 136] | 9.5 [8.2 ; 10.8] | 123 [105 ; 143] | 11.8 [10.8 ; 13] | 157 [142 ; 177] | 11.7 [11 ; 12.5] | 161 [148 ; 176] | <0.0001 |
| Others*** | 1.5 [1.3 ; 2] | 97 [86 ; 114] | 1.4 [1.1 ; 1.7] | 111 [84 ; 173] | 1.5 [1.4 ; 1.8] | 116 [98 ; 161] | 1.7 [1.6 ; 2] | 131 [114 ; 153] | 0.0001 |
| **Paramedical procedures** | **62.6 [56.9 ; 69.1]** | **1076 [968 ; 1207]** | **81.6 [54.5 ; 168.6]** | **1067 [851 ; 1368]** | **87.1 [72.9 ; 106.9]** | **1344 [1123 ; 1587]** | **85.8 [75 ; 98.2]** | **1329 [1187 ; 1503]** | <0.0001 |
| Nurse | 31.7 [27.6 ; 36.2] | 506 [427 ; 626] | 30.5 [21.8 ; 47.7] | 503 [334 ; 777] | 42.1 [33.4 ; 55.9] | 729 [563 ; 955] | 39 [32.5 ; 49.9] | 579 [477 ; 719] | <0.0001 |
| Physiotherapist | 30.3 [26.8 ; 35.2] | 483 [434 ; 541] | 50 [28.4 ; 144.5] | 464 [363 ; 605] | 44.4 [34 ; 60.2] | 542 [452 ; 641] | 45.8 [39.5 ; 54.7] | 636 [567 ; 714] | <0.0001 |
| Others† | 0.7 [0.4 ; 1] | 88 [68 ; 117] | 1.1 [0.5 ; 2.5] | 100 [61 ; 168] | 0.6 [0.3 ; 1.2] | 72 [50 ; 107] | 1.1 [0.6 ; 2.3] | 114 [89 ; 148] | 0.0038 |
| **Medications** | 147.1 [141.1 ; 153.1] | **1394 [1296 ; 1519]** | 156.9 [144.8 ; 169.5] | **1534 [1269 ; 2109]** | 179.6 [169.5 ; 189.9] | **1859 [1658 ; 2154]** | 203.6 [196 ; 212.2] | **2253 [2111 ; 2448]** | <0.0001 |
| Alimentary tract and metabolism | 19.6 [18.4 ; 20.8] | 124 [112 ; 141] | 20.2 [17.6 ; 23.2] | 108 [87 ; 137] | 22.3 [20.1 ; 24.9] | 148 [127 ; 179] | 24.1 [22.5 ; 26.2] | 156 [139 ; 179] | <0.0001 |
| Blood and blood forming organs | 9.4 [8.7 ; 10.4] | 100 [87 ; 122] | 10.5 [8.6 ; 13.4] | 117 [84 ; 179] | 11.8 [10.1 ; 14.3] | 191 [138 ; 299] | 10.7 [9.8 ; 11.9] | 137 [114 ; 170] | <0.0001 |
| Cardiovascular system | 16.2 [15.3 ; 17] | 134 [124 ; 143] | 16.3 [14.4 ; 18.3] | 123 [106 ; 142] | 18.3 [16.8 ; 20.1] | 139 [124 ; 155] | 20 [18.8 ; 21.2] | 172 [159 ; 186] | <0.0001 |
| Dermatologicals | 3.4 [3 ; 4] | 7 [6 ; 8] | 4.5 [3 ; 10.8] | 9 [6 ; 16] | 3.6 [3.1 ; 4.3] | 10 [7 ; 13] | 3.9 [3.5 ; 4.6] | 9 [7 ; 11] | 0.0004 |
| Genito-urinary system and sex hormones | 1.5 [1.1 ; 2.6] | 10 [8 ; 14] | 1.2 [0.9 ; 1.7] | 11 [7 ; 18] | 1.4 [1 ; 1.8] | 8 [6 ; 11] | 1.4 [1.2 ; 1.6] | 11 [8 ; 13] | 0.2982 |
| Systemic hormonal preparations | 5.5 [4.9 ; 6.4] | 20 [16 ; 28] | 6.5 [5.5 ; 7.8] | 18 [14 ; 23] | 7.5 [6.5 ; 8.6] | 38 [24 ; 72] | 8.8 [7.9 ; 9.8] | 31 [26 ; 48] | <0.0001 |
| Antiinfectives for systemic use | 7.8 [7.2 ; 8.4] | 77 [60 ; 113] | 9.4 [8.3 ; 10.9] | 76 [60 ; 113] | 10.8 [9.6 ; 12.1] | 131 [89 ; 252] | 12.5 [11.5 ; 13.9] | 135 [110 ; 176] | <0.0001 |
| Antineoplastic and immunomodulating agents | 0.3 [0.2 ; 0.5] | 72 [41 ; 127] | 0.6 [0.4 ; 1.1] | 176 [22 ; 920] | 0.4 [0.2 ; 0.7] | 142 [54 ; 489] | 0.5 [0.3 ; 0.7] | 86 [44 ; 188] | 0.2171 |
| Musculo-skeletal system | 3 [2.7 ; 3.5] | 23 [17 ; 35] | 2.8 [2.2 ; 3.8] | 13 [9 ; 18] | 2.8 [2.4 ; 3.3] | 18 [14 ; 30] | 3.7 [3.3 ; 4.1] | 30 [22 ; 46] | <0.0001 |
| Nervous system | 51.8 [48.6 ; 54.8] | 206 [182 ; 242] | 50.4 [45.1 ; 56.9] | 192 [147 ; 302] | 58.8 [53.7 ; 64.8] | 210 [182 ; 251] | 64 [60.1 ; 67.9] | 210 [189 ; 238] | <0.0001 |
| Antiparasitic products, insecticides and repellents | 0.1 [0.1 ; 0.1] | 4 [1 ; 19] | 0.1 [0 ; 0.2] | 1 [0 ; 1] | 0.1 [0.1 ; 0.2] | 1 [0 ; 1] | 0.1 [0.1 ; 0.1] | 2 [0 ; 13] | 0.3572 |
| **Respiratory system** | 25.1 [23.5 ; 26.6] | **476 [422 ; 559]** | 30 [27.1 ; 33.8] | **571 [441 ; 982]** | 36.7 [33.8 ; 40.1] | **673 [584 ; 885]** | 49 [46.6 ; 52] | **1137 [1030 ; 1284]** | <0.0001 |
| Sensory organs | 2.8 [2.5 ; 3.2] | 39 [27 ; 58] | 3.7 [2.9 ; 4.8] | 44 [23 ; 94] | 4.4 [3.6 ; 5.4] | 52 [32 ; 93] | 4.2 [3.8 ; 4.8] | 44 [29 ; 72] | <0.0001 |
| Various | 0.2 [0.2 ; 0.3] | 28 [9 ; 118] | 0.1 [0.1 ; 0.2] | 6 [3 ; 11] | 0.2 [0.2 ; 0.3] | 8 [6 ; 11] | 0.2 [0.1 ; 0.2] | 8 [6 ; 13] | 0.1744 |
| Medical deductible | 0.2 [0.1 ; 0.2] | 4 [3 ; 8] | 0.1 [0.1 ; 0.3] | 2 [1 ; 3] | 0.1 [0.1 ; 0.3] | 5 [2 ; 15] | 0.2 [0.1 ; 0.3] | 4 [3 ; 8] | 0.0546 |
| Dispensing honorary | 0 [0 ; 0] | 70 [65 ; 75] | 0 [0 ; 0] | 69 [58 ; 80] | 0 [0 ; 0] | 85 [75 ; 95] | 0 [0 ; 0] | 79 [73 ; 85] | 0.0073 |
| **Medical Device** | **10.4 [9.7 ; 11.2]** | **1078 [992 ; 1175]** | **10.7 [9.2 ; 12.5]** | **983 [799 ; 1226]** | **12.4 [11.2 ; 13.7]** | **1199 [1043 ; 1375]** | **14.7 [13.7 ; 15.8]** | **1410 [1285 ; 1543]** | <0.0001 |
| Respiratory assistance devices, home oxygen therapy | 2.5 [2.2 ; 2.8] | 540 [478 ; 612] | 1.8 [1.4 ; 2.5] | 419 [312 ; 575] | 2.5 [2.1 ; 3] | 616 [506 ; 768] | 3 [2.6 ; 3.3] | 689 [604 ; 783] | <0.0001 |
| Aerosol generator device | 1.6 [1.4 ; 1.9] | 42 [36 ; 50] | 2.1 [1.7 ; 2.7] | 56 [43 ; 75] | 2.8 [2.4 ; 3.3] | 69 [56 ; 85] | 3.5 [3.2 ; 4] | 78 [70 ; 90] | <0.0001 |
| Others | 6.3 [5.8 ; 6.8] | 497 [448 ; 555] | 6.7 [5.6 ; 8] | 508 [404 ; 654] | 7 [6.2 ; 7.9] | 514 [433 ; 615] | 8.2 [7.5 ; 8.9] | 642 [570 ; 724] | <0.0001 |
| **Transportation** | 3.9 [3.6 ; 4.3] | 444 [393 ; 528] | 3.6 [3.1 ; 4.2] | 362 [301 ; 444] | 4.1 [3.7 ; 4.7] | 457 [386 ; 653] | 4.3 [4 ; 4.7] | 507 [447 ; 614] | **<0.0001** |
| Ambulance | 2.6 [2.4 ; 2.9] | 299 [265 ; 366] | 2.6 [2.2 ; 3] | 262 [218 ; 330] | 2.7 [2.4 ; 3.1] | 323 [260 ; 525] | 2.9 [2.7 ; 3.2] | 350 [308 ; 410] | 0.0065 |
| light medical vehicles | 0.6 [0.5 ; 0.7] | 37 [26 ; 56] | 0.4 [0.3 ; 0.8] | 21 [12 ; 39] | 0.7 [0.5 ; 1] | 45 [28 ; 94] | 0.6 [0.5 ; 0.8] | 38 [28 ; 52] | 0.0117 |
| Taxis | 0.6 [0.5 ; 0.8] | 72 [48 ; 117] | 0.6 [0.3 ; 0.9] | 58 [32 ; 127] | 0.6 [0.5 ; 0.9] | 63 [43 ; 102] | 0.7 [0.6 ; 0.9] | 97 [61 ; 200] | 0.0030 |
| Others‡ | 0.1 [0.1 ; 0.1] | 36 [27 ; 50] | 0.1 [0 ; 0.1] | 21 [11 ; 40] | 0.1 [0 ; 0.1] | 25 [16 ; 42] | 0.1 [0 ; 0.1] | 22 [16 ; 31] | 0.6665 |
| **Productivity loss** | 2.6 [1.7 ; 3.9] | 289 [216 ; 385] | 1 [0.3 ; 4.4] | 286 [134 ; 547] | 1.8 [0.8 ; 4.1] | 190 [113 ; 325] | 2.3 [1.3 ; 3.8] | 249 [172 ; 350] | 0.9765 |
| Dailly allowance | 2.6 [1.6 ; 3.9] | 80 [52 ; 118] | 1 [0.3 ; 3.3] | 36 [8 ; 133] | 1.8 [0.9 ; 4.4] | 64 [28 ; 153] | 2.3 [1.3 ; 3.8] | 69 [40 ; 118] | 0.9517 |
| Invalidity pension | 0 [0 ; 0] | 209 [146 ; 308] | 0 [0 ; 0] | 249 [120 ; 473] | 0 [0 ; 0] | 126 [60 ; 234] | 0 [0 ; 0] | 180 [116 ; 282] | 0.6928 |
| **Global** |  | **15303 [14335 ; 16445]** |  | **17327 [15015 ; 20364]** |  | **18390 [16817 ; 20147]** |  | **18188 [17159 ; 19391]** | **<0.0001** |

Supplementary Table 4: Details of medical costs according to short acting beta agonist dispensation in patients who died from asthma over 12 months prior to death. CI : Confident Interval ; Other inpatient care* : Hospital reserved drugs, hospital outpatient department, emergency, dialysis.. , ** Other consultations: Teleconsultations, mark-up, flat-rate participation; Other paramedical acts†: pedicure-podiatrist, speech therapists, orthoptists, midwives, thermal baths ...; Other medical acts***: anesthesia, surgery, dental, obstetrics … ; Other transportation‡ : personal vehicles, public transport…

|  | 0 canister (N=1218) | | [1; 2] canisters (N=559) | | [3; 5] canisters (N=479) | | [6; 12] canisters (N=643) | | >12 canisters (N=930) | |  | |
| --- | --- | --- | --- | --- | --- | --- | --- | --- | --- | --- | --- | --- |
|  | Frequency | Cost | Frequency | Cost | Frequency | Cost | Frequency | Cost | Cost | Frequency | | P-value |
|  | Mean  [95 CI] | Mean  [95 CI] | Mean  [95 CI] | Mean  [95 CI] | Mean  [95 CI] | Mean  [95 CI] | Mean  [95 CI] | Mean  [95 CI] | Mean  [95 IC] | Mean  [95 IC] | |  |
| **Inpatient stays** |  | **10271 [9361 ; 11519]** |  | **10930 [9492 ; 12819]** |  | **9615 [8371 ; 11212]** |  | **12582 [11253 ; 14305]** |  | **12727 [11568 ; 13992]** | | 0.0001 |
| Medicine, Surgery, Obstetrics | 1.7 [1.6 ; 2] | 7019 [6517 ; 7657] | 2 [1.7 ; 3] | 7875 [6798 ; 9648] | 1.7 [1.5 ; 2] | 7170 [6294 ; 8125] | 2.3 [2 ; 3.5] | **9382 [8528 ; 10482]** | 3 [2.4 ; 3.9] | **10009 [9147 ; 10950]** | | 0.0001 |
| *Respiratory system* | 0.6 [0.5 ; 0.6] | 2610 [2319 ; 3034] | 0.6 [0.5 ; 0.7] | 2885 [2350 ; 4243] | 0.7 [0.7 ; 0.9] | **3002 [2521 ; 3669]** | 0.9 [0.8 ; 1] | **4029 [3473 ; 4710]** | 1.1 [1 ; 1.2] | **4760 [4219 ; 5428]** | | 0.0001 |
| Asthma related | 0.3 [0.3 ; 0.4] | 1118 [947 ; 1352] | 0.4 [0.3 ; 0.5] | 1688 [1262 ; 2625] | 0.4 [0.4 ; 0.5] | 1560 [1244 ; 1915] | 0.5 [0.5 ; 0.6] | 1939 [1630 ; 2306] | 0.7 [0.6 ; 0.8] | 2693 [2356 ; 3107] | | 0.0001 |
| Others | 0.3 [0.2 ; 0.3] | 1492 [1248 ; 1852] | 0.2 [0.2 ; 0.3] | 1198 [918 ; 1620] | 0.3 [0.2 ; 0.4] | 1442 [1137 ; 1910] | 0.3 [0.3 ; 0.4] | 2091 [1662 ; 2680] | 0.3 [0.3 ; 0.4] | 2068 [1717 ; 2553] | | 0.0172 |
| *Not related with the Respiratory system* | 1.2 [1 ; 1.4] | 4409 [3979 ; 4897] | 1.3 [1 ; 2.5] | 4990 [4205 ; 6655] | 1 [0.8 ; 1.2] | 4168 [3553 ; 4878] | 1.4 [1.1 ; 2.6] | 5353 [4644 ; 6199] | 1.9 [1.4 ; 2.9] | 5249 [4660 ; 5987] | | 0.0314 |
| Rehabilitation | 0.5 [0.3 ; 0.9] | 2515 [1929 ; 3711] | 0.4 [0.3 ; 0.6] | 2702 [1946 ; 3858] | 0.3 [0.3 ; 0.4] | 1590 [1207 ; 2196] | 0.4 [0.3 ; 0.5] | 2642 [1937 ; 3632] | 0.3 [0.3 ; 0.4] | 1953 [1573 ; 2648] | | 0.9419 |
| Home Care Service | 0.1 [0.1 ; 0.2] | 398 [221 ; 719] | 0.2 [0.1 ; 0.6] | 185 [67 ; 565] | 0.1 [0.1 ; 0.3] | 143 [55 ; 341] | 0.1 [0 ; 0.3] | 209 [81 ; 610] | 0.1 [0.1 ; 0.2] | 376 [193 ; 743] | | 0.2961 |
| Others* | 0 [0 ; 0] | 339 [219 ; 552] | 0 [0 ; 0] | 168 [128 ; 340] | 0 [0 ; 0] | 712 [264 ; 2501] | 0 [0 ; 0] | 349 [203 ; 779] | 0 [0 ; 0.1] | 389 [249 ; 696] | | 0.0001 |
| Outpatient pneumologist consultation | 0.1 [0.1 ; 0.1] | 3 [2 ; 4] | 0.1 [0.1 ; 0.2] | 4 [2 ; 6] | 0.1 [0.1 ; 0.1] | 2 [1 ; 3] | 0.1 [0.1 ; 0.2] | 4 [3 ; 6] | 0.3 [0.2 ; 0.3] | 7 [6 ; 9] | | <0.0001 |
| **Consultation** | **13.9 [12.8 ; 15.4]** | **345 [327 ; 363]** | **16.6 [14.5 ; 21.5]** | **391 [361 ; 428]** | **15.1 [13.8 ; 16.8]** | **413 [383 ; 449]** | **15.3 [13.9 ; 17.8]** | **391 [365 ; 422]** | **17.1 [15.8 ; 19.3]** | **447 [421 ; 474]** | | **0.0001** |
| General practitioner | 12.6 [11.5 ; 14.1] | 256 [241 ; 270] | 14.8 [12.7 ; 20.5] | 286 [264 ; 319] | 13.4 [12.2 ; 15.2] | 300 [277 ; 330] | 13.7 [12.3 ; 16] | 287 [267 ; 311] | 15.1 [13.9 ; 17.5] | 328 [308 ; 349] | | 0.0001 |
| Specialist | 1.4 [1.2 ; 1.6] | 38 [34 ; 44] | 1.8 [1.4 ; 2.9] | 47 [38 ; 63] | 1.7 [1.4 ; 2] | 48 [40 ; 59] | 1.6 [1.3 ; 2.1] | 46 [38 ; 61] | 1.9 [1.6 ; 2.4] | 58 [49 ; 73] | | 0.0081 |
| Pneumologist | 0.1 [0.1 ; 0.1] | 2 [2 ; 3] | 0.1 [0.1 ; 0.2] | 3 [2 ; 5] | 0.1 [0.1 ; 0.2] | 3 [2 ; 5] | 0.2 [0.1 ; 0.2] | 4 [3 ; 5] | 0.2 [0.1 ; 0.2] | 4 [3 ; 6] | | <0.0001 |
| Others** | 0 [0 ; 0] | 51 [47 ; 55] | 0 [0 ; 0] | 58 [51 ; 69] | 0 [0 ; 0] | 65 [56 ; 79] | 0 [0 ; 0] | 57 [51 ; 69] | 0 [0 ; 0] | 61 [56 ; 68] | | 0.0138 |
| **Medical procedures** | **12.5 [11.7 ; 13.4]** | **278 [257 ; 308]** | **13.9 [12.8 ; 15.6]** | **309 [279 ; 345]** | **13.5 [12.3 ; 14.9]** | **321 [284 ; 384]** | **13.2 [12.1 ; 14.5]** | **321 [287 ; 365]** | **14.1 [13.2 ; 15.3]** | **347 [319 ; 387]** | | **0.0001** |
| Imaging | 1 [0.9 ; 1.1] | 51 [45 ; 57] | 1.3 [1.1 ; 1.5] | 68 [58 ; 79] | 1.2 [1 ; 1.4] | 62 [53 ; 77] | 1.2 [1 ; 1.3] | 60 [52 ; 73] | 1.2 [1.1 ; 1.3] | 62 [55 ; 70] | | 0.0430 |
| Laboratory tests | 10.3 [9.6 ; 11.2] | 132 [122 ; 144] | 10.9 [9.9 ; 12] | 144 [129 ; 162] | 10.8 [9.8 ; 12.1] | 141 [126 ; 160] | 10.5 [9.5 ; 11.6] | 141 [127 ; 165] | 11 [10.2 ; 11.9] | 154 [141 ; 169] | | 0.0021 |
| Others*** | 1.2 [1.1 ; 1.3] | 96 [82 ; 116] | 1.7 [1.3 ; 3.6] | 97 [82 ; 117] | 1.4 [1.2 ; 1.7] | 118 [94 ; 167] | 1.5 [1.3 ; 1.9] | 120 [98 ; 157] | 2 [1.7 ; 2.7] | 132 [114 ; 157] | | 0.0001 |
| **Paramedical procedures** | **69.9 [61.9 ; 82.6]** | **1094 [977 ; 1236]** | **70.2 [59.5 ; 84.5]** | **1078 [914 ; 1269]** | **63.3 [52.4 ; 78.3]** | **1117 [928 ; 1369]** | **82.2 [65.3 ; 120.4]** | **1237 [1047 ; 1509]** | **85.3 [74.6 ; 98.7]** | **1398 [1215 ; 1617]** | | **0.0074** |
| Nurse | 33.3 [27.9 ; 39.4] | 509 [426 ; 614] | 35.2 [27.2 ; 47.6] | 465 [354 ; 604] | 26.9 [21.2 ; 34.2] | 483 [351 ; 709] | 39.4 [30.5 ; 54.9] | 615 [458 ; 852] | 39.5 [33 ; 48.2] | 687 [559 ; 915] | | 0.0001 |
| Physiotherapist | 35.7 [30.1 ; 44] | 486 [432 ; 552] | 34.1 [27.9 ; 42.1] | 530 [437 ; 634] | 35.8 [28.1 ; 48] | 551 [452 ; 666] | 41.7 [29.6 ; 77.1] | 540 [460 ; 645] | 45.2 [38.2 ; 55.3] | 600 [528 ; 691] | | 0.2226 |
| Others† | 0.9 [0.6 ; 1.3] | 99 [75 ; 137] | 0.9 [0.4 ; 1.7] | 83 [56 ; 132] | 0.6 [0.2 ; 1.7] | 82 [55 ; 126] | 1.2 [0.4 ; 3.5] | 82 [57 ; 118] | 0.7 [0.4 ; 1.2] | 111 [82 ; 149] | | 0.8020 |
| **Medications** | **139.3 [132.5 ; 146.3]** | **1352 [1238 ; 1523]** | **162.6 [153 ; 172.7]** | **1625 [1443 ; 1933]** | **167.2 [156.2 ; 179.3]** | **1620 [1455 ; 1916]** | **171.9 [162.8 ; 181.9]** | **1677 [1525 ; 1912]** | **215 [206 ; 225]** | **2417 [2221 ; 2673]** | | **0.0001** |
| Alimentary tract and metabolism | 19.1 [17.7 ; 20.5] | 125 [107 ; 153] | 20.6 [18.6 ; 22.9] | 135 [113 ; 176] | 20.9 [18.8 ; 23.5] | 129 [107 ; 157] | 21.2 [19.4 ; 23.4] | 134 [116 ; 155] | 25.4 [23.5 ; 27.9] | 157 [141 ; 177] | | 0.0001 |
| Blood and blood forming organs | 9.9 [8.9 ; 11.1] | 105 [89 ; 129] | 10.6 [9.2 ; 12.6] | 137 [106 ; 196] | 12.4 [10.6 ; 15] | 152 [116 ; 226] | 10.3 [9 ; 12] | 141 [108 ; 238] | 9.5 [8.4 ; 10.9] | 123 [95 ; 164] | | 0.1423 |
| Cardiovascular system | 16.6 [15.5 ; 17.7] | 136 [125 ; 148] | 18.6 [17 ; 20.3] | 149 [135 ; 165] | 18.4 [16.5 ; 20.4] | 135 [119 ; 153] | 17 [15.5 ; 18.4] | 145 [130 ; 161] | 18.7 [17.5 ; 20] | 162 [149 ; 179] | | 0.0005 |
| Dermatologicals | 3.1 [2.8 ; 3.5] | 7 [6 ; 9] | 3.6 [3.1 ; 4.2] | 7 [6 ; 9] | 4.9 [3.7 ; 7.2] | 12 [9 ; 18] | 3.3 [2.8 ; 3.9] | 7 [5 ; 8] | 4.2 [3.5 ; 5.9] | 9 [8 ; 12] | | 0.0004 |
| Genito-urinary system and sex hormones | 1.3 [1 ; 1.5] | 11 [9 ; 17] | 2.4 [1.3 ; 9.3] | 12 [8 ; 20] | 1.4 [1.1 ; 1.9] | 9 [6 ; 13] | 1.3 [1 ; 1.7] | 10 [7 ; 15] | 1.2 [1 ; 1.4] | 8 [6 ; 11] | | 0.7800 |
| Systemic hormonal preparations | 4.6 [4.1 ; 5.5] | 15 [11 ; 23] | 5.9 [5 ; 7.3] | 29 [19 ; 53] | 6.7 [5.7 ; 8] | 25 [16 ; 58] | 7.4 [6.5 ; 8.5] | 29 [21 ; 54] | 10.3 [9.2 ; 12] | 37 [30 ; 60] | | 0.0001 |
| Antiinfectives for systemic use | 8.2 [7.5 ; 9.1] | 90 [67 ; 137] | 8.7 [7.9 ; 9.8] | 89 [61 ; 194] | 10 [9 ; 11.1] | 76 [65 ; 95] | 10.1 [8.8 ; 12.2] | 101 [75 ; 159] | 12.3 [11.2 ; 13.7] | 142 [114 ; 217] | | 0.0000 |
| Antineoplastic and immunomodulating agents | 0.4 [0.3 ; 0.6] | 118 [56 ; 279] | 0.4 [0.2 ; 0.7] | 93 [20 ; 465] | 0.3 [0.2 ; 0.6] | 115 [41 ; 294] | 0.4 [0.2 ; 0.6] | 41 [22 ; 85] | 0.5 [0.3 ; 0.7] | 94 [41 ; 287] | | 0.7846 |
| Musculo-skeletal system | 2.7 [2.4 ; 3.1] | 28 [19 ; 44] | 2.9 [2.5 ; 3.6] | 15 [11 ; 19] | 2.9 [2.4 ; 3.5] | 13 [9 ; 18] | 3.5 [2.9 ; 4.8] | 22 [16 ; 42] | 3.8 [3.4 ; 4.3] | 30 [22 ; 47] | | 0.0001 |
| Nervous system | 49.8 [46.4 ; 53.9] | 172 [154 ; 195] | 58.4 [53.2 ; 63.7] | 217 [185 ; 260] | 55.5 [50.3 ; 62] | 216 [175 ; 287] | 56.3 [51.5 ; 61.7] | 195 [168 ; 237] | 64.6 [60.4 ; 69.7] | 249 [211 ; 327] | | 0.0001 |
| Antiparasitic products, insecticides and repellents | 0.1 [0 ; 0.1] | 3 [0 ; 11] | 0.1 [0 ; 0.1] | 1 [0 ; 1] | 0.1 [0 ; 0.2] | 0 [0 ; 1] | 0.1 [0.1 ; 0.2] | 10 [0 ; 60] | 0.2 [0.1 ; 0.2] | 1 [1 ; 1] | | 0.1311 |
| **Respiratory system** | 19.7 [18.2 ; 21.3] | 417 [369 ; 501] | 26.6 [23.9 ; 29.4] | 600 [489 ; 808] | 29.3 [26.7 ; 32] | 548 [494 ; 637] | 36.6 [33.9 ; 39.3] | 732 [621 ; 947] | 60.1 [57.2 ; 63.7] | 1256 [1125 ; 1460] | | 0.0001 |
| Sensory organs | 3.4 [3 ; 3.9] | 48 [33 ; 74] | 3.5 [2.9 ; 4.3] | 46 [25 ; 89] | 3.7 [3 ; 4.6] | 37 [20 ; 82] | 3.8 [3.1 ; 4.5] | 25 [18 ; 39] | 3.6 [3.2 ; 4.2] | 50 [31 ; 85] | | 0.1735 |
| Various | 0.2 [0.1 ; 0.2] | 9 [6 ; 14] | 0.2 [0.1 ; 0.3] | 10 [7 ; 20] | 0.3 [0.2 ; 0.6] | 74 [7 ; 402] | 0.2 [0.1 ; 0.2] | 7 [5 ; 15] | 0.2 [0.1 ; 0.3] | 10 [6 ; 26] | | 0.7173 |
| Medical  deductible | 0.1 [0.1 ; 0.2] | 4 [2 ; 8] | 0.2 [0.1 ; 0.3] | 6 [3 ; 16] | 0.2 [0.1 ; 0.3] | 3 [2 ; 5] | 0.2 [0.1 ; 0.3] | 5 [2 ; 14] | 0.2 [0.1 ; 0.3] | 4 [2 ; 6] | | 0.2714 |
| Dispensing honorary | 0 [0 ; 0] | 66 [61 ; 72] | 0 [0 ; 0] | 77 [69 ; 88] | 0 [0 ; 0] | 75 [67 ; 87] | 0 [0 ; 0] | 72 [64 ; 80] | 0 [0 ; 0] | 85 [78 ; 93] | | 0.0003 |
| **Medical Device** | 9.6 [8.8 ; 10.5] | 957 [862 ; 1059] | 11.3 [10.2 ; 12.6] | 1181 [1009 ; 1361] | 11.7 [10.3 ; 13.3] | 1134 [952 ; 1354] | 12.1 [11 ; 13.4] | 1195 [1055 ; 1356] | 15.8 [14.6 ; 17.2] | 1525 [1373 ; 1692] | | <0.0001 |
| Respiratory assistance devices, home oxygen therapy | 2.2 [1.9 ; 2.6] | 474 [407 ; 555] | 2.3 [1.9 ; 2.7] | 503 [407 ; 626] | 2.5 [2 ; 3] | 533 [424 ; 679] | 2.5 [2.1 ; 3] | 575 [473 ; 702] | 3.4 [2.9 ; 3.9] | 820 [715 ; 938] | | <0.0001 |
| Aerosol generator device | 1 [0.8 ; 1.3] | 23 [19 ; 30] | 1.8 [1.5 ; 2.3] | 38 [31 ; 49] | 1.9 [1.6 ; 2.5] | 49 [38 ; 77] | 2.7 [2.3 ; 3.2] | 73 [61 ; 93] | 4.7 [4.2 ; 5.3] | 111 [98 ; 125] | | 0.0065 |
| Others | 6.3 [5.7 ; 7] | 460 [410 ; 518] | 7.2 [6.3 ; 8.2] | 640 [531 ; 786] | 7.3 [6.3 ; 8.5] | 551 [450 ; 687] | 6.9 [6.1 ; 7.8] | 548 [474 ; 643] | 7.7 [7 ; 8.5] | 594 [521 ; 675] | | 0.1613 |
| **Transportation** | 3.8 [3.5 ; 4.2] | 404 [357 ; 460] | 3.9 [3.5 ; 4.5] | 444 [359 ; 640] | 3.8 [3.3 ; 4.5] | 389 [336 ; 476] | 3.9 [3.5 ; 4.5] | 451 [376 ; 579] | 4.6 [4.2 ; 5.1] | 578 [486 ; 729] | | **0.1885** |
| Ambulance | 2.8 [2.5 ; 3.1] | 303 [272 ; 346] | 2.4 [2.1 ; 2.7] | 293 [231 ; 491] | 2.7 [2.3 ; 3.2] | 286 [242 ; 363] | 2.6 [2.3 ; 3] | 285 [245 ; 363] | 3 [2.7 ; 3.4] | 379 [321 ; 502] | | 0.0361 |
| light medical vehicles | 0.5 [0.4 ; 0.7] | 22 [17 ; 30] | 0.7 [0.5 ; 1] | 48 [28 ; 119] | 0.5 [0.4 ; 0.7] | 26 [17 ; 43] | 0.6 [0.5 ; 0.8] | 54 [35 ; 98] | 0.7 [0.6 ; 0.9] | 44 [30 ; 71] | | 0.0804 |
| Taxis | 0.5 [0.4 ; 0.6] | 55 [36 ; 98] | 0.7 [0.5 ; 1.1] | 60 [41 ; 90] | 0.6 [0.4 ; 0.8] | 51 [33 ; 90] | 0.6 [0.4 ; 0.9] | 79 [42 ; 169] | 0.8 [0.6 ; 1.1] | 128 [73 ; 249] | | 0.6123 |
| Others‡ | 0.1 [0 ; 0.1] | 23 [17 ; 33] | 0.1 [0.1 ; 0.3] | 43 [23 ; 86] | 0.1 [0 ; 0.1] | 26 [16 ; 43] | 0.1 [0.1 ; 0.1] | 33 [21 ; 60] | 0.1 [0 ; 0.1] | 27 [19 ; 37] | | 0.0033 |
| **Productivity loss** | 1.1 [0.6 ; 2.1] | 198 [135 ; 289] | 1.1 [0.4 ; 3.6] | 236 [127 ; 443] | 1.6 [0.6 ; 4.1] | 169 [82 ; 334] | 3 [1.5 ; 5.8] | 330 [214 ; 555] | 4.2 [2.7 ; 6.4] | 363 [266 ; 493] | | **0.0034** |
| Dailly allowance | 1.1 [0.5 ; 2.2] | 33 [17 ; 71] | 1.1 [0.4 ; 3.3] | 41 [12 ; 148] | 1.6 [0.6 ; 4.2] | 55 [16 ; 161] | 3 [1.5 ; 5.7] | 95 [54 ; 166] | 4.2 [2.7 ; 6.5] | 129 [80 ; 204] | | 0.0874 |
| Invalidity pension | 0 [0 ; 0] | 165 [102 ; 252] | 0 [0 ; 0] | 195 [92 ; 391] | 0 [0 ; 0] | 114 [44 ; 234] | 0 [0 ; 0] | 236 [127 ; 457] | 0 [0 ; 0] | 234 [157 ; 349] | | <0.0001 |
| **Global** |  | 14899 [13837 ; 16309] |  | 16194 [14620 ; 18373] |  | 14777 [13244 ; 16653] |  | 18183 [16683 ; 19998] |  | 19802 [18516; 21281] | | <0.0001 |

Supplementary Table 5: number of patients died from asthma among all the patients who died between 2013 and 2017 in France

|  | AsthmA | All | Mortality rate |
| --- | --- | --- | --- |
| 2013 | 755 | 567078 | 0.13% |
| 2014 | 748 | 557081 | 0.13% |
| 2015 | 788 | 591809 | 0.13% |
| 2016 | 766 | 592074 | 0.13% |
| 2017 | 772 | 604299 | 0.13% |
| Total | 3829 | 2912341 | 0.13% |
