# Supplementary material for: The economic burden of asthma prior to death: a nationwide descriptive study
Source: Front Public Health. 2024 Feb 19;12:1191788. doi: 10.3389/fpubh.2024.1191788 (PMC10909909; doi:10.3389/fpubh.2024.1191788)
Supplement: Supplementary file 2 [file Table_2.DOCX]

Supplementary materials

Medical costs

Costs were recorded and analysed from the French health insurance perspective. Direct medical (i.e., hospitalisations, medical and paramedical acts, medications, consultations, medical devices), and non-medical (i.e., transportation) costs as well as costs related to absence from the workplace (i.e., daily allowance) were included in the analysis.

Inpatient stays were assessed using French Disease Related Groups (DRG) plus additional charges if applicable (i.e., days in intensive care unit, expensive drugs, or medical devices). Costs related to the emergency room (ER) were assessed using the emergency flat rate if the patient was not admitted to a medical unit after passing through the ER. The costs of outpatient care provided in town or in a public or private healthcare establishment (outside inpatient stays) were identified and assessed on the basis of the prices applied by the French Health Insurance. The tariffs of the General Nomenclature of Professional Acts (NGAP), Common Classification of Medical Acts (CCAM), and the Nomenclature of Acts in Medical Biology (NABM) were applied.

Transportation costs were assessed taking into account the distance between the patient's place of residence and their place of care, the mode of transportation used, and the level of care.

Costs related to absence from the workplace were assessed using daily allowances given by the French Health Insurance for the compensation of lost earnings due to short-term absence.
